# Epigenetic and genetic alterations and their influence on gene regulation in chronic lymphocytic leukemia

**DOI:** 10.1186/s12864-017-3617-6

**Published:** 2017-03-16

**Authors:** Di Huang, Ivan Ovcharenko

**Affiliations:** Computational Biology Branch, National Center for Biotechnology Information, National Library of Medicine, National Institutes of Health, Bethesda, MD 20892 USA

**Keywords:** DNA methylation, Regulatory elements, Genetic mutation, Transcription factor binding site, Genome-wide association study

## Abstract

**Background:**

To understand the changes of gene regulation in carcinogenesis, we explored signals of DNA methylation – a stable epigenetic mark of gene regulatory elements — and designed a computational model to profile loss and gain of regulatory elements (REs) during carcinogenesis. We also utilized sequencing data to analyze the allele frequency of single nucleotide polymorphisms (SNPs) and detected the cancer-associated SNPs, i.e., the SNPs displaying the significant allele frequency difference between cancer and normal samples.

**Results:**

After applying this model to chronic lymphocytic leukemia (CLL) data, we identified REs differentially activated (dREs) between normal and CLL cells, consisting of 6,802 dREs gained and 4,606 dREs lost in CLL. The identified regulatory perturbations coincide with changes in the expression of target genes. In particular, the genes encoding DNA methyltransferases harbor multiple lost-in-cancer dREs and zero gained-in-cancer dREs, indicating that the damaged regulation of these genes might be one of the key causes of tumor formation. dREs display a significantly elevated density of the genome-wide association study (GWAS) SNPs associated with CLL and CLL-related traits. We observed that most of dRE GWAS SNPs associated with CLL and CLL-related traits (83%) display a significant haplotype association among the identified cancer-associated alleles and the risk alleles that have been reported in GWAS. Also dREs are enriched for the binding sites of the well-established B-cell and CLL transcription factors (TFs) NF-kB, AP2, P53, E2F1, PAX5, and SP1. We also identified CLL-associated SNPs and demonstrated that the mutations at these SNPs change the binding sites of key TFs much more frequently than expected.

**Conclusions:**

Through exploring sequencing data measuring DNA methylation, we identified the epigenetic alterations (more specifically, DNA methylation) and genetic mutations along non-coding genomic regions CLL, and demonstrated that these changes play a critical role in carcinogenesis through damaging the regulation of key genes and alternating the binding of key TFs in B and CLL cells.

**Electronic supplementary material:**

The online version of this article (doi:10.1186/s12864-017-3617-6) contains supplementary material, which is available to authorized users.

## Background

Cancer, a leading cause of death worldwide [1], is a major focus of biological and clinical research. Dramatic phenotypic alterations in cancer cells have often been attributed to gene mutation and gene regulatory variation [2]. In the last decade, evidence has been accumulating that the malfunction of gene regulatory elements, such as promoters, enhancers, etc., makes a substantial contribution to cancer initiation and progression. For example, the promoter inactivation of von Hippel-Lindau (VHL), leading to the silencing of this gene, has been reported as a biomarker of renal cancer [3]. Similarly, in many cancers, the transcription of cyclin-dependent kinase inhibitor 2A (CDKN2A), an important tumor suppressor gene, has been found to be terminated after the chromatin blocking of its promoter region [4]. Also, the disruption of super-enhancers plays a key role in inhibiting the oncogene MYC in multiple myeloma [5]. More recently, aberrant DNA methylation along super-enhancers has been reported in a broad spectrum of cancers, such as breast, colon, lung cancer [6].

To delineate the activity alteration of regulatory elements (REs) during carcinogenesis, the signals of epigenetic marks are commonly measured and compared between cancer and normal cells [7]. DNA methylation, predominantly occurring at the 5’ position of the cytosine in CpG dinucleotides, is a stable epigenetic mark that can be combined with other epigenetic modifiers, such as Histone 3 lysine 4 trimethylation (H3K4me3), for defining the function of the DNA. DNA methylation preliminarily affects the activity of regulatory elements, prompting research into how DNA methylation alters gene regulation. Since the original report in 1983 that DNA methylation is substantially decreased in tumor tissues, aberrant DNA methylation has been well-established as a signature in cancer [8–10]. Global hypomethylation of repetitive DNAs elements has been found to be responsible for promoting multiple cancers, such as inducing the overexpression of oncogenes in leukemia cells [11], silencing the tumor suppressor genes in colorectal cancer [12], and enhancing the chromatin instability in lymphoma [13].

With the knowledge that de-methylation is strongly correlated with activation of regulatory elements [14], we developed a computational model, in which a genome-wide methylation profile was analyzed to map REs in cancer and normal cells. The comparison between these RE maps in turn established differentially-activated REs (dREs), including dREs gained and lost during cancer development. We tested this model on chronic lymphocytic leukemia (CLL), due to its relatively abundant data resources, and observed that the gained and lost dREs were enriched in the neighborhood of up- and down-regulated genes during CLL carcinogenesis. The genes encoding transcription repressors and DNA methyltransferases have multiple lost dREs in their loci, suggesting an important role for these genes in maintaining normal B-cells and initiating CLL development. Also, dREs are enriched for the GWAS SNPs associated with CLL or, more broadly, cancer traits. CLL genetic mutations, i.e., the substitution of wild - type alleles with CLL-susceptible alleles, are associated with a change in binding of major B-cell TFs. In this study, we identified epigenetic and genetic changes during carcinogenesis and evaluated the impact of these changes on gene regulation.

## Methods

### Data processing of reduced representative bisulfite sequencing (RRBS)-seq profiling

We analyzed the genome-wide methylation profiles from 32 B cells of 32 chronic lymphocytic leukemia (CLL) patients and 10 normal CD19+ B cells [15] (which have been deposited to Gene Expression Omnibus GSE66121 by the authors of the referenced study). Methylation levels of CpG sites were measured using reduced representative bisulfite sequencing (RRBS)-seq.

We downloaded the raw RRBS-seq reads to establish the methylation profiles and detect the genetic mutations in CLL. We established a workflow to analyze these raw sequence data (Additional file 1: Figure S1). Bismark [16] coupled with Bowtie2 [17] was used to align the raw reads to the human genome with the settings “-q --phred64-quals -n 1 -l 40”. The alignment results, i.e., one sam file per sample, were transformed into bam files using the samtools (“samtools view -bT”) [18]. The bam files were used as input to BisSNP [19] to calculate the methylation levels of CpG sites and to call genotypes. The parameters for BisSNP were set as default, i.e., -maxQ 40, -stand_call_conf 8, -stand_emit_conf 0, -mmq 30, -mbq 0. Only SNPs with the minor allele frequency (MAF) > 0.01 in 1000 Genomes Project [20] were used to run BisSNP.

### Mapping consensus dREs in a sample class

We combined methylation profiles of all samples from a class (i.e., either CLL or control) to obtain consensus dREs. In a methylation profile of a sample, we excluded CpG sites with less than five aligned reads. To account for the variable numbers of reads across samples, we penalized each sample according to its total number of aligned reads. That is, the combined number of reads at a CpG site (e.g., *k*) was calculated as

1 $$ R( k)=\frac{{\displaystyle {\sum}_{i\in samples\  under\  consideration}}{w}_i{r}_{i k}}{{\displaystyle {\sum}_{i\in samples\  under\  consideration}}{w}_i}, $$

where *r*_*ik*_ is the number of the reads at the site *k* from the sample *i. w*_*i*_, the weight of the sample *i*, is determined as the reciprocal of the total number of the aligned reads in the sample *i*. After replacing *r*_*ik*_ with *mr*_*ik*_, the number of the methylated reads, in eq. (1), we obtained the combined number of methylated reads at *k*. After collecting these numbers, we had a combined methylation profile for each tested sample class. We then applied MethylSeekR [21] to each combined methylation profile with the setting of chr.sel = chr2, meth.cutoff = 0.5 and nCpG.cutoff = 3. At the end, we established a map of consensus dREs, together with sREs and hiMRs, in each sample class.

To categorize dREs based on their genomic location, we employed the annotatePeaks.pl script from HOMER with default settings. The obtained gained dREs, lost dREs and sREs, with average lengths of 660, 814, and 1094 bp, have the average CpG density of 2.4, 3.9 and 5.6 CpGs per 100 bp, respectively.

### Hierarchal clustering and PCA

After filtering out CpGs with less than five aligned reads, we used the classic hierarchal clustering algorithm to analyze the similarity of methylation profiles from different samples (32 CLL cells and 10 control B-cells). For this purpose, we employed the MATLAB function “linkage” to build a hierarchal clustering tree using the distance of “Euclidean” and the method of “ward.” The constructed tree was then visualized using the MATLAB function “dendrogram” with the default settings.

We also used principal component analysis (PCA) to visualize the distribution of samples. PCA was conducted by using the MATLAB function “princomp” with the default settings.

### Alignment of human and mouse genomes

To map genomic regions (i.e., dREs, sREs and hiMRs) from the human to the mouse genome, we used the software “bnMapper” (available at https://bitbucket.org/james_taylor/bx-python/wiki/bnMapper). The pair-wise genome alignment (chain file) between the mouse and human required by bnMapper was downloaded from the UCSC Genome browser. bnMapper was run with the setting “--gap 20 --threshold 0.1”. A human genomic region was considered as conserved between human and mouse when the aligned sequence was longer than 20 bps.

### Repeat composition along dREs

We used the repeat tables downloaded from the UCSC Genome browser to investigate the repeat content of dREs. Given a group of dREs, the fraction of these regions covered by repetitive elements was calculated. Similarly, the repeat composition of sREs and hiMRs was estimated and was used as a baseline to evaluate the enrichment of repeats in dREs.

### Enrichment of dREs in loci of genes differentially expressed in CLL

The RNA-seq profiles of the 32 CLL samples, together with five normal B-cell samples (of which two samples were also included in the methylation data), were downloaded from Gene Expression Omnibus (accession number GSE66117). To avoid unreliable RNA-seq measurements, we filtered out genes with very low expression, i.e., those for which the average expression was less than 0.1 in either CLL or normal B-cells. For each of the remaining genes, the fold change of its expression in CLL was then calculated as the ratio of the average expression in CLL to that in normal B-cells. Ranking the genes based on their expression fold-change, we identified genes up- and down-regulated in CLL by selecting a percentile of top differentially regulated genes.

Following a general rule, we assigned a genomic region (either RE or hiMR) to the gene with the closest transcription start site (TSS). Given gained (or lost) dREs and a group of genes (say *R* and *G*, respectively), we identified dREs linked to any given gene, and calculated the fraction of these dREs from all dREs associated with the genes having reliable RNA-seq measurements (denoted as *fract*(*R*, *G*)). Using the sREs (represented by *S*) as background, we evaluated the enrichment of *G* in the surrounding of *R* as the ratio of *fract*(*R*, *G*) to *fract*(*S*, *G*). The significance of this enrichment was measured under a binomial test.

### Functional analysis of dREs and genes

We used the Genomic Regions Enrichment of Annotations Tool (GREAT, available at http://bejerano.stanford.edu/great/public/html/) [22] to examine the function of dREs with the whole human genome as the background. Also, the Database for Annotation, Visualization and Integrated Discovery (DAVID, available at https://david.ncifcrf.gov/) [23] was used to estimate the function of a set of genes with the whole list of human genes as the background.

### Distribution analysis of dREs

Given a class of dREs, we calculated the distance from each dRE to its nearest within-class neighbor and then computed the distribution of these distances. Through randomly shuffling class labels among dREs, sREs and hiMRs, we generated a background class and assessed the distribution of within-class distances in the background class. We generated 1,000 background classes independently and used the average of their within-class distributions as background for statistical analysis. Similarly, we built the distribution of cross-class distances of gained dREs to their nearest lost dREs and compared this distribution with the background estimated the same way as in the case for within-class computations.

The bimodal distribution of within-class distances among lost dREs (Additional file 1: Figure S2) implies that parts of lost dREs are clustered close to each other (the distance of <10 kb). To investigate the function of these lost dREs, we identified the lost dREs with the distances to their nearest within-class neighbor less than 10 kb. We used GREAT to evaluate the function of these lost dREs (see Functional analysis of dREs).

### GWAS analysis of dREs

We downloaded the NHGRI GWAS Catalog in April 2015 [24]. For each GWAS SNP, we identified all SNPs in a tight LD with the GWAS SNP (*r* ^2^ > 0.8 and *distance* < 500 *kb*) based on at least one population from the 1000 Genomes Project (CEU, YRI and CHB/JPN) by using SNAP [25]. After that, we linked these tight-LD SNPs to the corresponding traits. At the end, we had 1,759 GWAS traits associated with 324,454 SNPs.

Considering that 54% of the traits are linked to a small number of SNPs, i.e., less than five tagged SNPs, we agglomerated similar traits together to obtain reliable statistical results. For example, we identified the GWAS SNPs associated with the lymphoma traits (but not CLL), and marked them as “lymphoma”. Similarly, we built SNP categories for “CLL” and “cancer” due to their immediate and close relevance with CLL. Finally, the GWAS SNPs not included in these categories were marked as “irrelevant” and were used as the baseline of our statistical analysis.

To evaluate the association between a class of dREs (e.g., gained dREs) and a GWAS trait, we identified all SNPs from the 1000 Genomes Project in gained dREs. After that, we counted among these SNPs the ones that have been associated with a given trait. This count measures the overlap between the given GWAS trait and gained dREs. To examine the significance of this count, we adopted a permutation strategy. We randomly shuffled class labels among dREs, sREs and hiMRs, and counted the SNPs linked to the tested trait in the randomly-labeled gained dREs. After repeating this process 1,000 times, we examined the probability of randomly-labeled gained dREs displaying a higher number of given-trait-associated SNPs than the gained dREs. This probability measures the significance of the association between the gained dREs and the tested given trait.

### Identification of SNPs and their alleles associated with CLL

We investigated RRBS reads at SNP positions. For those SNPs that were not polymorphic in the set of RRBS reads, we dubbed them non-assayed if they overlapped with less than 10 reads or non-mutated otherwise.

Given a SNP, we compared its allele frequencies in the population of CLL samples with those of the control population using a binomial test. Given a SNP and its allele *k*, we have

2 $$ Pr\left( X>{n}_{k, c}\right)=1-{\displaystyle \sum_{i=0}^{n_{k, c}}}\left(\begin{array}{c}\hfill {n}_c\hfill \\ {}\hfill i\hfill \end{array}\right){p_{k, n}}^i{\left(1-{p}_{k, n}\right)}^{\left({n}_c- i\right)}, $$

where *n*_*k*,*c*_ is the occurrence count of *k* in the CLL samples, and *n*_*c*_ is the summation of the occurrence count of all alleles in the CLL samples. *p*_*k*,*n*_ is the frequency of *k* in the control samples. We used the MATLAB function “binocdf” for this calculation. We also examined the significance of each diploid genotype state in CLL samples with reference to controls. The minimum of the *p* values (i.e., *Pr* s) of the alleles and genotype states measures the significance of genotypic difference between CLL and control. The nucleotide positions having *minimum of Prs* < 0.05 were marked CLL-associated SNPs. In this study, we detected 305 and 186 CLL-associated SNPs located in lost and gained dREs, respectively. Furthermore, for a CLL-associated SNP, the allele enriched in CLL was considered as the CLL-associated allele.

### Haplotype association between alleles

Given a CLL-associated SNP (i.e., *m*) and a GWAS tag SNP (i.e., *m*_*tag*) of which the risk allele has been reported in GWAS studies, we explored the 1000 Genomes Project genotype data to examine haplotype association between the CLL-associated allele (represented as 1|*m*) and the risk allele at the tag SNP (say 1|*m*_*tag*). In detail, we downloaded the genotype data of *m* and *m*_*tag* from the 1000 Genome Project for all populations and built a 2 × 2 contingency table composed by *D*_11_, *D*_12_, *D*_21_, and *D*_22_ (Additional file 1: Figure S3). *D*_11_ is the number of the chromosomes genotyped as (1|*m*, 1|*m*_*tag*). This rule was applied to define *D*_12_, *D*_21_, and *D*_22_ with 2|*m* representing the non CLL-susceptible allele(s) at *m* and 2|*m*_*tag* representing the non-risk allele(s) at *m*_*tag*. With the built contingency table, the haplotype association of (1|*m*, 1|*m*_*tag*_) was tested using Fisher’s exact test and the odd ratio (*OR*) was estimated as

3 $$ OR=\frac{D_{11}{D}_{22}}{D_{12}{D}_{21}}. $$

### TFBS representation and enrichment along dREs

We used the TRANScription FACtor (TRANSFAC) version 2010.3 [26] and JASPAR [27] databases of TFBS. We scanned dREs sequences using position weight matrices (PWMs) from these two databases using Find Individual Motif Occurrences (FIMO) [28] with the default settings.

Given a dREs, we randomly sampled the human genome to obtain 10 control sequences with matching GC content, repeat density, and sequence length. TFBS enrichment in the dREs was calculated as the ratio of a TFBS density in dREs to counterpart in control sequences.

### Binding affinity changes at CLL-associated allele substitution positions

Given a CLL-associated SNP, we regarded the CLL-associated allele as the mutant allele (MU), and the other allele as the wild - type allele (WT). To estimate the impact of the CLL-associated alleles in lost dREs, we evaluated the fraction of the TFBSs disrupted after replacing WT with MU alleles (Additional file 1: Figure S4). For a TFBS *t*, we have

4 $$ Fract\left( lost\Big| t\right)=\frac{the\  number\  of\  TFBSs\  in\  WT\  but\  not\  in\  MU\ }{the\  number\  of\  TFBSs\  in\  WT}=\frac{N\left( t\Big| WT,\  not\  MU\right)}{N\left( t\Big| WT\right)}. $$

To evaluate the significance of *Fract*(*lost*|*t*), we first generated control sequences for lost dREs. Given a CLL-associated SNP *s*, we scanned the lost dRE sequence carrying *s* and randomly chose *N* nucleotide positions having the matched WT allele (i.e., the reference alleles for non-mutated positions) with *s*. For a background position, its *MU* sequence was constructed by replacing the WT allele with the MU allele of *s*. In this study, we set *N* = 30, i.e., we had 30 background positions for each CLL-associated SNP. The significance of *Fract*(*lost*|*t*) was then estimated using a binomial distribution,

5 $$ Pr\left( X> N\left( t\Big| WT,\  not\  MU\right)\right)=1-{\displaystyle \sum_{i=0}^{N\left( t\Big| WT,\  not\  MU\right)}}\left(\begin{array}{c}\hfill N\left( t\Big| WT\right)\hfill \\ {}\hfill i\hfill \end{array}\right){p_t}^i{\left(1-{p}_t\right)}^{\left({n}_c- i\right)}, $$

where *p*_*t*_ is *Fract*(*lost*|*t*) in controls.

On the other hand, the impact of the CLL-associated alleles in gained dREs is the significance of the number of TFBSs generated after substituting WT with MU alleles. For a TFBS *t*, we have

6 $$ Fract\left( gained\Big| t\right)=\frac{the\  number\  of\  TFBSs\  in\  MU\  but\  not\  in\  WT\ }{the\  number\  of\  TFBSs\  in\  MU}=\frac{N\left( t\Big| MU,\  not\  WT\right)}{N\left( t\Big| MU\right)}. $$

The significance of *Fract*(*gained*|*t*) was estimated as

7 $$ Pr\left( X> N\left( t\Big| MU,\  not\  WT\right)\right)=1-{\displaystyle \sum_{i=0}^{N\left( t\Big| MU,\  not\  WT\right)}}\left(\begin{array}{c}\hfill N\left( t\Big| MU\right)\hfill \\ {}\hfill i\hfill \end{array}\right){p_t}^i{\left(1-{p}_t\right)}^{\left({n}_c- i\right)}, $$

where *p*_*t*_ is *Fract*(*gained*|*t*) in the background positions generated using the strategy for lost dRE SNPs.

## Results

### Methylation of non-promoter CpG sites is informative for distinguishing CLL from control

**Fig. 1:**
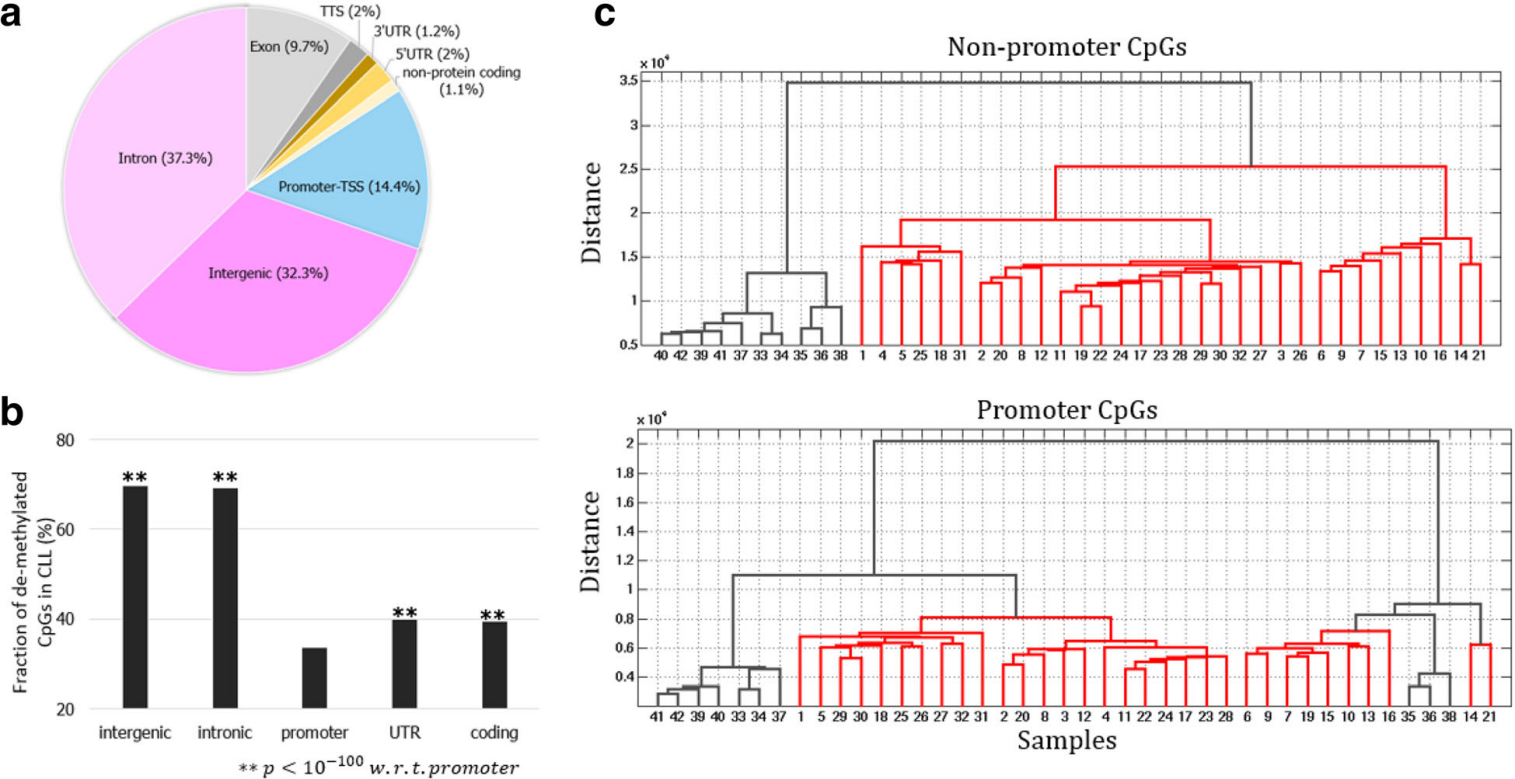
Methylation of CpG sites in CLL and control samples. **a** The pie chart shows the genomic distribution of CpG sites assayed in RRBS-seq. **b** Fractions of the CpG sites de-methylated in CLL along different genomic regions are compared. To control the noise introduced during data preparation and process, only the CpGs having a considerable methylation change, i.e., the methylation difference between CLL and control is greater than 0.3, were considered here. **c** The results of a classic hierarchical algorithm show that methylation of the CpGs located in gene regulatory loci can distinguish CLL (in red color) from control (black)

CLL is a biologically and clinically heterogeneous disease, in which the genomic and genetic alterations leading its progression and development have yet to be fully understood [29]. We started our analysis with the genome-wide DNA methylation profiles previously established by applying reduced representation bisulfite sequencing (RRBS) to CD19+ B-cells from 32 CLL patients and 10 normal B-cell samples [15] (see Methods). Thirty-two percent of three million assayed CpG sites reside in CpG islands (Additional file 1: Figure S5). With the aim of understanding proximal and distant gene regulation mechanisms during carcinogenesis, we focused on gene regulatory elements (consisting of promoter, intronic and intergenic elements), excluding other genomic segments (such as exons). Eighty-four percent of assayed CpG sites reside along gene regulatory elements (Fig. 1a and Additional file 1: Figure S6, see Methods). To delineate the contribution of promoters and distal regulatory elements (such as enhancers and silencers), we further divided these regulatory elements into two parts – promoter and non-promoter sequences (i.e., intronic and intergenic genomic loci).

Next, we directly compared the CpG methylation profiles in CLL with those of normal B-cells. To control the noise introduced during data generation and processing, we focused on the CpGs having a considerable methylation change (i.e., the difference of methylation level between CLL and normal cells is greater than 0.3), denoted as methyl-change CpGs. Thus, every methyl-change CpG is either highly methylated in normal cells but not CLL (we refer to this class as de-methylated in CLL) or, in the opposite case, highly methylated in CLL but not in normal (we refer to those as highly methylated in CLL). After focusing on regulatory elements (i.e., non-promoter, intronic and intergenic elements), we observed that about 70% of non-promoter methyl-change CpGs are de-methylated in CLL (69.1% and 69.7% for intergenic and intronic, respectively), significantly higher than that in promoters (33.7%, binomial test *p* < 10^− 100^ intergenic/introns vs. promoters, Fig. 1b), which demonstrates that non-promoter CpGs predominantly lose methylation, while promoter CpGs become predominantly methylated. This is in accordance with the report that de-methylation is widespread in intergenic and intronic regions in cancer cells [30]. Promoters display the smallest fraction of methyl-change CpGs among all genomic regions (Fig. 1b), reflecting that the promoters are more likely to become methylated than other genomic regions in carcinogenesis [31].

We applied a classic hierarchical clustering algorithm to the CpGs methylation profiles in regulatory elements (see Methods). Using the methylation levels of CpGs located in non-promoter gene regulatory loci, all CLL samples, being clustered into a homogeneous group, were distinguished from normal samples (Fig. 1c). On the other hand, the methylation signals of promoter CpGs could be used to cluster the majority but not all CLL samples together (Fig. 1c). These findings are further supported when using principal component analysis (PCA) to visualize the distribution of CLL and control samples (see Methods, Additional file 1: Figure S6). Collectively, the nucleobase-resolution methylation profiles of CpGs in gene regulatory elements (including promoter and non-promoter elements) contain sufficient information to discriminate CLL from control samples. Especially, non-promoter CpGs are capable of distinguishing two sample classes better than promoter CpGs (Fig 1c and Additional file 1: Figure S6).

### Mapping consensus REs in CLL and control

As methylation is highly correlated over short genomic distances and the methylation change at individual CpG sites correlates with chromatin accessibility and transcription factor association of the flanking context [32], our next step was to expand base-resolution methylation levels to local methylation states. By connecting multiple adjacent CpGs (i.e., at least three CpGs in this study) with similar methylation levels [21], we identified the de-methylated regions and marked those located in gene regulatory loci as activated REs (see Methods and Additional file 1: Figure S1). Our assumption was that methylation change corresponds to the change in the activity of a RE—as methylated REs are likely inactive, long spans of CpG de-methylation in CLL likely correspond to REs that have been inactive in normal cells, but have been activated in CLL. Throughout the rest of the manuscript, we use these differential methylation data in reference to REs that are active in either normal or CLL cells.

**Fig. 2:**
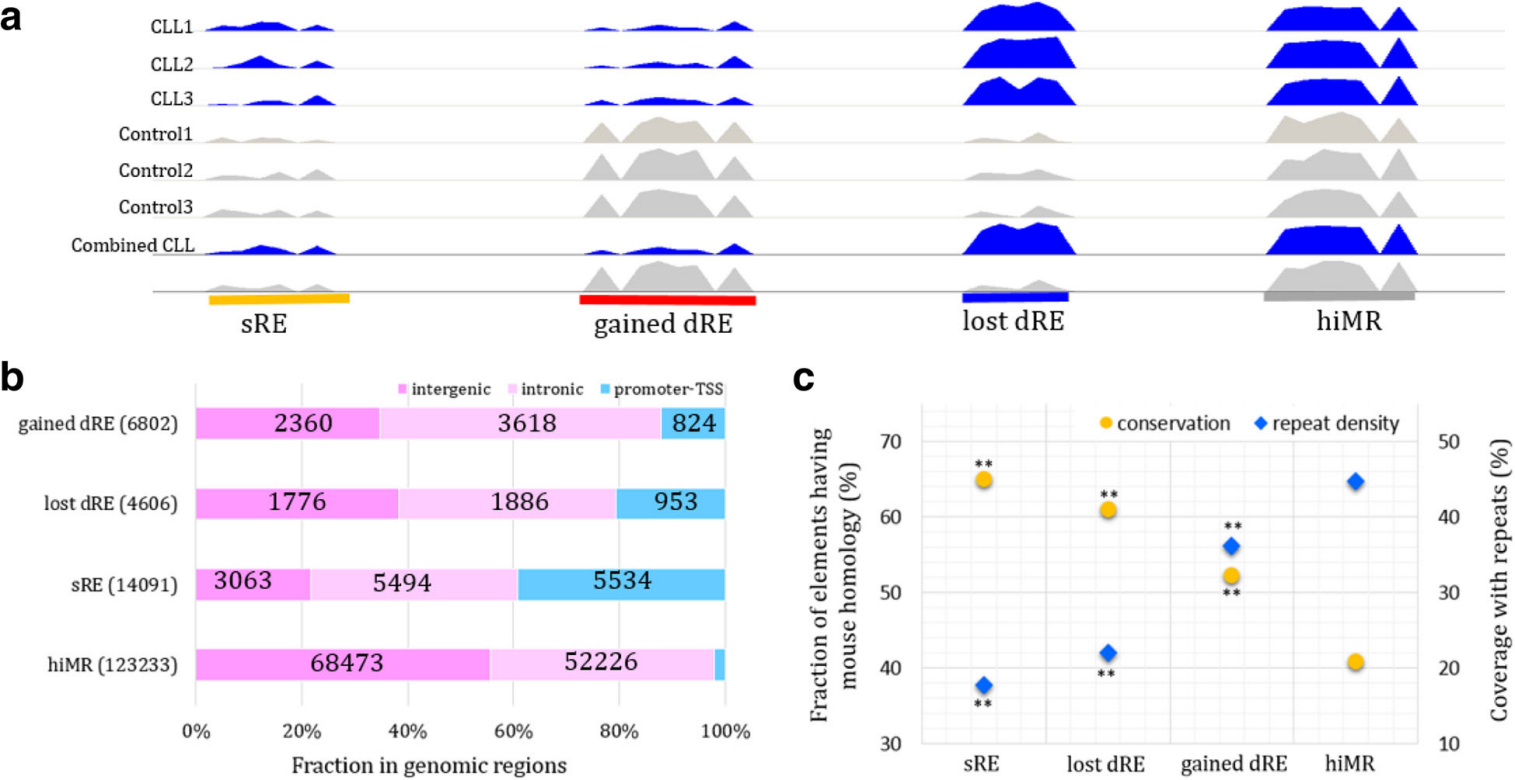
Mapping the consensus dREs in CLL and normal samples. **a** The definitions of consensus dRE and hiMR are depicted. **b** Stacked bar graph shows the genomic distribution of REs. REs are categorized into gained dREs, lost dREs, and shared REs (sREs) according to their activity states in CLL with respect to normal samples. **c** Fractions of the conserved and repeat nucleotide positions in the dRE sequences are plotted. A position is regarded as conserved when it shows the identical nucleotide with the aligned mouse genome position

To obtain consistent methylation signals in a sample class, we adopted a read-number-based normalization strategy to average methylation signals across samples in a class. We then used the averaged methylation profile to predict consensus REs for a tested sample class (Fig. 2a). By comparing the landscapes of REs in CLL and control, we identified dREs which were further subcategorized into dREs gained and dREs lost during CLL development (Fig. 2a, see Methods). We also identified REs shared by CLL and control (sREs) and the regions highly-methylated in both CLL and control (hiMRs), which were used as a background reference in the following analysis. In total, we identified 6,802 gained dREs, 4,606 lost dREs, 14,091 sREs and 123,233 hiMRs (Fig. 2b). In additional file 1: Figure S7, examples of dREs and sREs are given.

dREs occupy non-promoter regions (i.e., intronic and intergenic genomic loci) more often than sREs (88% gained and 79% lost dREs vs. 60% sREs, binomial test *p* < 10^− 100^, Fig. 2b), which is in line with the preceding finding that non-promoter CpGs display larger methylation changes than promoter CpGs (Fig. 1b), and suggests a pronounced role of enhancer changes during CLL development.

In addition, gained dREs in 22.6% of promoters and 3.6% of non-promoters overlap CpG islands (CGIs), which is significantly lower than their lost dRE and sRE counterparts (44% and 58.7% of promoters lost dRE and sRE, respectively, and 18% and 28.7% of non-promoters lost dRE and sRE, respectively; *p* < 10^− 100^, Additional file 1: Figure S8, see Methods). The significant depletion of CpG islands (CGIs) along the gained dREs coincides with the report that DNA methylation in tumors is higher within CGIs but is lower outside of CGIs [33].

### dREs of different categories show distinct evolutionary conservation

We assessed evolutionary conservation of dREs by aligning their human and mouse counterparts (see Methods). First of all, more than half of sREs and dREs have mouse orthologues (65% of sREs, 60.9% of lost dREs and 52.3% of gained dREs), which is significantly higher than that of hiMRs (40.8%, binomial test *p* < 10^− 100^, Fig. 2c). This elevated evolutionary conservation is suggestive of molecular maintenance of dRE and sRE functionality. Moreover, dREs show higher sequence divergence than sREs (60.9% and 52.3% v.s. 65%, binomial test *p* < 10^− 100^), indicating the propensity of functional change of dREs during CLL development.

In addition, forty-four percent of hiMR sequence nucleotides are DNA sequence repeats, which is consistent with DNA repeats and repeat-derived regions spanning about half of the human genome [34] (Fig. 2c and Additional file 1: Figure S9). The low repeat density of dREs and sREs (17.65%, 22.14%, and 36.15% in sRE, lost dRE, and gained dRE sequences, respectively) is in agreement with a previous observation of decreased repeat content in regulatory elements [35] and correlates with their elevated evolutionary conservation. As compared with sREs and lost dREs, the gained dREs show the higher content of all classes of retrotransposons (Additional file 1: Figure S9, S10 and Additional file 2: Table S1), which supports the implication of retrotransposons in cancer initiation [36].

### Gain and loss of dREs positively correlate with the change of target gene expression

**Fig. 3:**
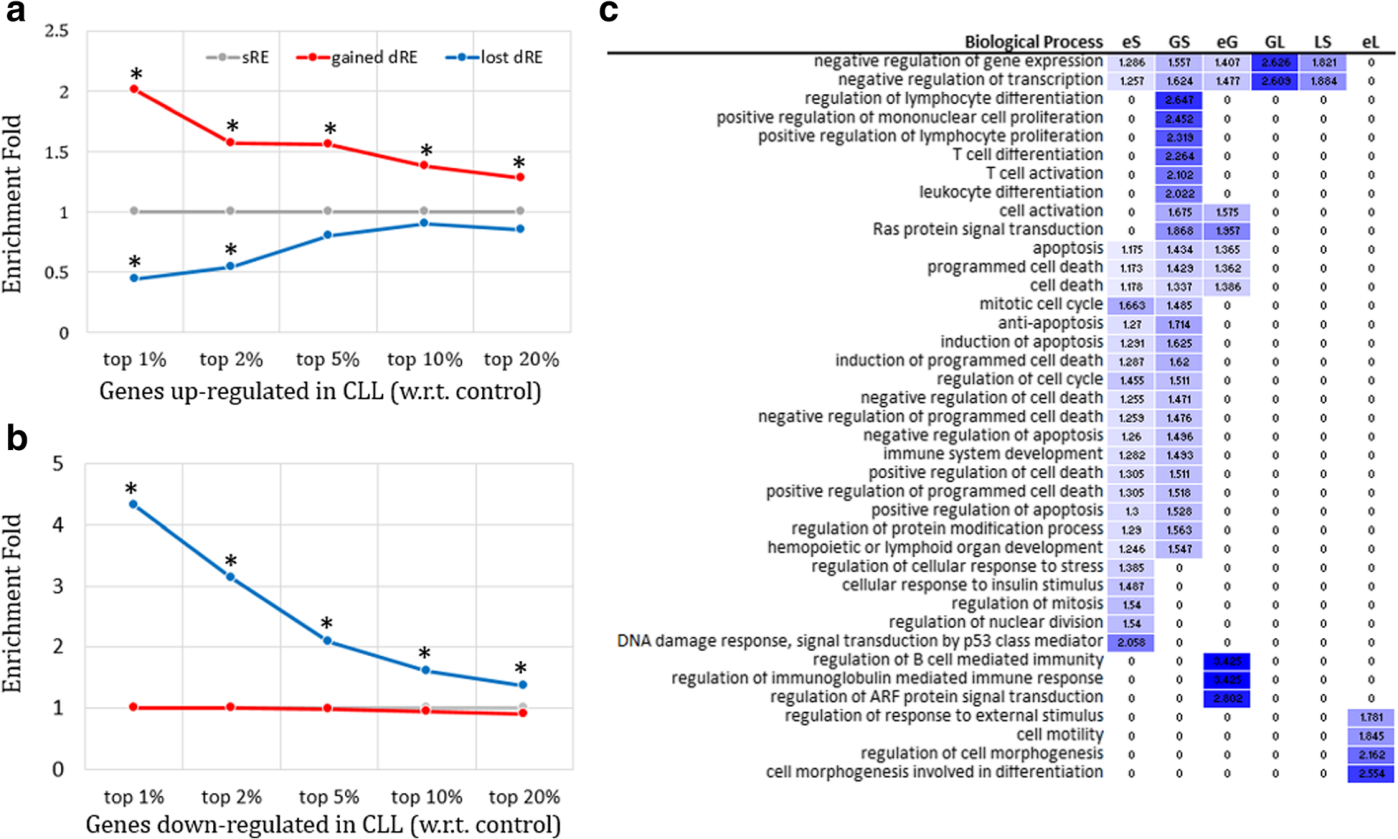
Functional analysis of dREs. Enrichments of the dREs are plotted for the genes (**a**) up-regulated in CLL and (**b**) down-regulated in CLL. The * indicates that binomial test *p* < 0.001. **c** Biological process association is compared across different gene groups. Human genes are categorized in light of the distribution of dREs and sREs. GS is a set of the genes harboring the gained dREs and sREs in their neighborhood. Similarly, GL represents the genes connected with both the gained and lost dREs, while LS is the genes linked to the lost dREs and sREs. eG represents the genes associated exclusively with the gained dREs, while eS and eL are the genes having only the shared dRE(s) and the lost dRE(s), respectively, in their neighborhood. The values presented in this heatmap are the enrichment fold with respect to all human genes. Zeros indicate insignificant enrichment (i.e, *p* > 0.001) in the corresponding cases

To gain insight into the phenotypic impact of dRE alteration, we explored gene expression data of the tested CLL and control samples (see Methods). Gained dREs are highly enriched in the neighborhood of the genes up-regulated in CLL samples. For instance, in the neighborhood of the top 1% of CLL-up-regulated genes are gained dREs enriched by two times as compared to sREs (binomial test *p* = 7 × 10^− 11^, Fig. 3a and Additional file 2: Table S2). Similarly, significant enrichment of gained dREs was also observed in regions around the top 2%, 5% and 10% of genes highly up-regulated in CLL (*p* < 10^− 7^). By contrast, the lost dREs are pronouncedly depleted in the neighborhood of the CLL-up-regulated genes (*p* = 6 × 10^− 3^, Fig. 3b and Additional file 2: Table S2). In addition, lost and gained dREs shows opposite distribution trends in the neighborhood of CLL-down-regulated genes. That is, lost dREs are overrepresented in the loci of CLL-down-regulated genes (*p* < 10^− 10^). All these observations support that the loss and gain of REs in CLL are strongly correlated with the changes in gene expression – the upregulated genes witness the gain of REs while the downregulated genes are associated with the loss of REs, suggesting that the change in gene regulation might be one of the key mechanisms of carcinogenesis.

Next, we binned human genes according to the presence of dREs and sREs in their neighborhood (see Methods). About 69% of the genes associated with the gained dREs (2,626/3,784) also harbor one or more sREs in their loci, while only 50% of the lost dRE genes (1,464/2,905) host sREs (Additional file 1: Figure S11). The genes linked to either the gained dREs or sREs or both are enriched with the genes participating in apoptosis, cell death and immunological process. All these biological processes are activated in normal B-cells and are impaired in cancer cells [37]. Also, genes exclusively linked to the lost dREs play a role in cell motility (Fig. 3c and Additional file 2: Table S3), and abnormal motility has been found in CLL cells [38]. Furthermore, the observation that the genes associated with both the gained dREs and sREs have a function in T cell differentiation and activation (Fig. 3c) partially explains the finding that T cell numbers are increased in most patients with CLL [39].

### Lost dREs cluster near the genes encoding DNA methyltransferases and transcription repressors

The analysis of the distribution of dREs revealed that the distances between two neighboring gained dREs (i.e., within-class distances between gained dREs) are significantly shorter than expected (Wilcoxon rank-sum test *p* < 10^− 16^, Additional file 1: Figure S3, see Methods). Similarly, the within-class distances of the lost dREs are much smaller than expected (*p* < 10^− 16^). The cross-class distance (i.e., the distance of a gained dRE to its nearest lost dRE) is longer than expected (*p* < 10^− 16^, Additional file 1: Figure S3). These findings show that dREs having the same activity likely cluster together, suggesting that the change of DNA activation occurs selectively, rather than randomly, along the human genome during CLL development. That is, certain genomic regions are subject to become activated (e.g., methylation decrease), while others tend to be de-activated (e.g., methylation increase).

As indicated by the bimodal distribution of within-class distance among lost dREs, there exist genomic regions having a high abundancy of lost dREs (Additional file 1: Figure S3). We hypothesized that the genes located near these regions play essential roles in maintaining normal B-cells, being buffered for regulatory alteration and thus requiring multiple lost dREs for their transcriptional disruption during CLL carcinogenesis. To examine this hypothesis, we detected the genes harboring multiple lost dREs in their loci (see Methods), and noticed that these genes are significantly associated with methyltransferase activity (enrichment fold = 2.06, *p* = 1.67 × 10^− 4^, Table 1). For example, the two DNA methyltransferase genes MGMT and DNMT1 harbor three lost dREs but no gained dREs in their loci. Transcriptional disruptions of these genes and inactivating mutations at their coding regions have been reported in leukemogenesis, such as acute myelogenous leukemia (AML) and CLL [40]. Besides, the genes regulating the binding of NFKB, such as P53 and hypoxia-inducible factor 1 (HIF1), host multiple lost dREs in their neighborhood. NFkB is a major TF in normal and CLL B-cells, of which the binding activity is altered during CLL development [41].

**Table 1:** Functional analysis of multi-lost dREs with respect to all lost dREs

Rank	Molecular function	*P*-value	Enrichment fold	Number of elements	
				multi-lost dREs	all dREs
1	NF-kappaB binding	1.67E-04	2.06	12	12
2	DNA-methyltransferase activity	1.67E-04	2.06	12	12
3	miRNA binding	3.46E-04	2.06	11	11
4	oxidoreductase activity, acting on NAD(P)H, oxygen as acceptor	3.46E-04	2.06	11	11
5	superoxide-generating NADPH oxidase activity	3.46E-04	2.06	11	11
6	peptidyl-histidine dioxygenase activity	7.16E-04	2.06	10	10
7	oxygen sensor activity	7.16E-04	2.06	10	10
8	peptidyl-asparagine 3-dioxygenase activity	7.16E-04	2.06	10	10
9	protein methyltransferase activity	7.16E-04	2.06	10	10
10	protein-lysine N-methyltransferase activity	7.16E-04	2.06	10	10

### CLL and CLL-related GWAS SNPs fall in dREs

**Fig. 4:**
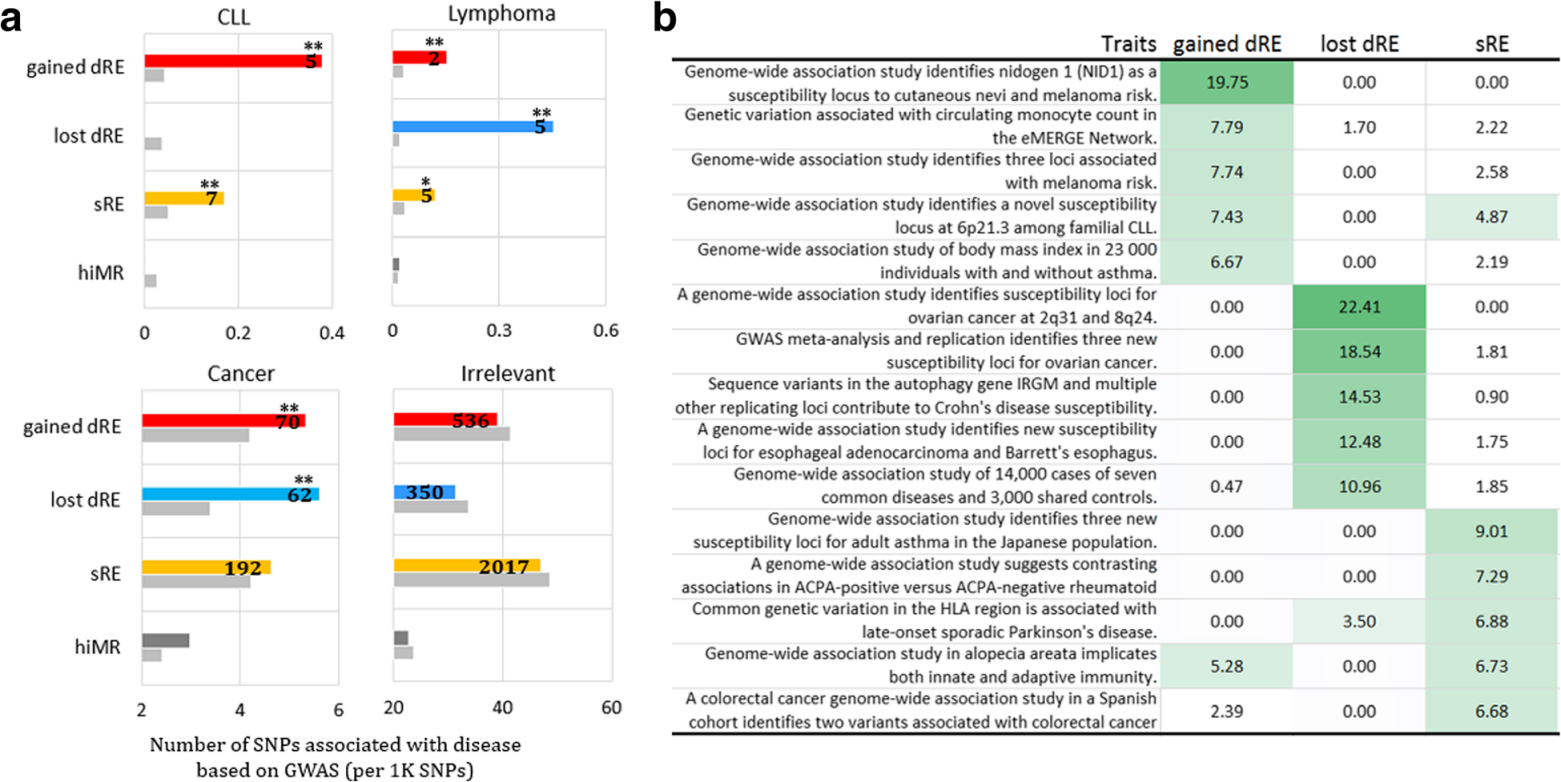
Association of dREs with GWAS traits. **a** The density of GWAS SNPs along the dREs is displayed for the agglomerated traits, including CLL, lymphoma and cancer. The GWAS SNPs not linked to these traits are marked as irrelevant and used as reference. The density of GWAS SNPs along the dREs is measured as the number of SNPs associated with a tested trait per 1,000 SNPs. The ** indicates that binomial test *p* < 1 × 10^− 5^. **b** Heatmap showing the SNP enrichment along dREs for individual GWAS traits. The enrichment is the ratio of the SNP density in the dREs to the expected density. In a cell, the shade of color correlates with the enrichment level, the darker green being higher enrichment. The white cells represent insignificant enrichment, i.e, *p* > 0.05

To address the phenotypic or pathological impact of dREs, we explored the results of GWAS. The NHGRI GWAS collection [24], in which approximately 200,000 SNPs are associated with 1,106 phenotypic or pathological traits, was used for this purpose. Overall, 3,262 GWAS SNPs or the SNPs located in tight - link disequilibrium blocks with GWAS SNPs (*r* ^2^ > 0.8) reside in dREs and sREs, of which 415, 608, and 2,239 SNPs are in the lost dREs, gained dREs, and sREs, respectively (see Methods). As more than half of GWAS SNP categories consist of less than five SNPs, we agglomerated the GWAS SNP categories linked with similar traits to generate broad SNP categories for CLL, lymphoma, and other cancers (such as melanoma, colorectal, ovarian and breast) to obtain reliable statistical estimates. These diseases were chosen due to their direct relevance to CLL. We labeled all GWAS SNPs not falling into any of these three broad categories as irrelevant and used them as baseline (see Methods). No enrichment of irrelevant SNPs in dREs suggests that our analysis strategy is able to address the ascertainment bias of GWAS SNPs (Fig. 4a and Additional file 2: Table S4). Our analysis revealed that each dRE group shows a distinct profile of GWAS traits. Gained and lost dREs are significantly enriched for the SNPs associated with CLL or, more broadly, lymphoma, (*p* < 5 × 10^− 5^, Fig. 4a and Additional file 2: Table S4). In addition, the dREs, rather than the sREs, are significantly enriched for cancer SNPs (*p* < 0.02), suggesting that dREs mutations are primarily susceptibility candidates for cancers, including haematological cancers. All dRE/sRE CLL and lyphoma SNPs are detailed in Additional file 1: Figure S12 and Additional file 1: Figure S13, respectively.

We also examined the association of dREs with individual GWAS traits (see Methods). The results consistently support the aforementioned functions of dREs (Fig. 3c), since the gained and lost dREs are strongly enriched for the SNPs linked to CLL and/or other cancers (*p* < 5 × 10^− 3^, Fig. 4b and Additional file 2: Table S5). Also, the sREs are remarkably linked to immunity-related traits, such as asthma and adaptive immunity (*p* < 1 × 10^− 8^), which is in line with the observations that sREs are significantly associated with T cell activation and differentiation (Fig. 3c).

### Examples of GWAS SNPs in dREs

**Fig. 5:**
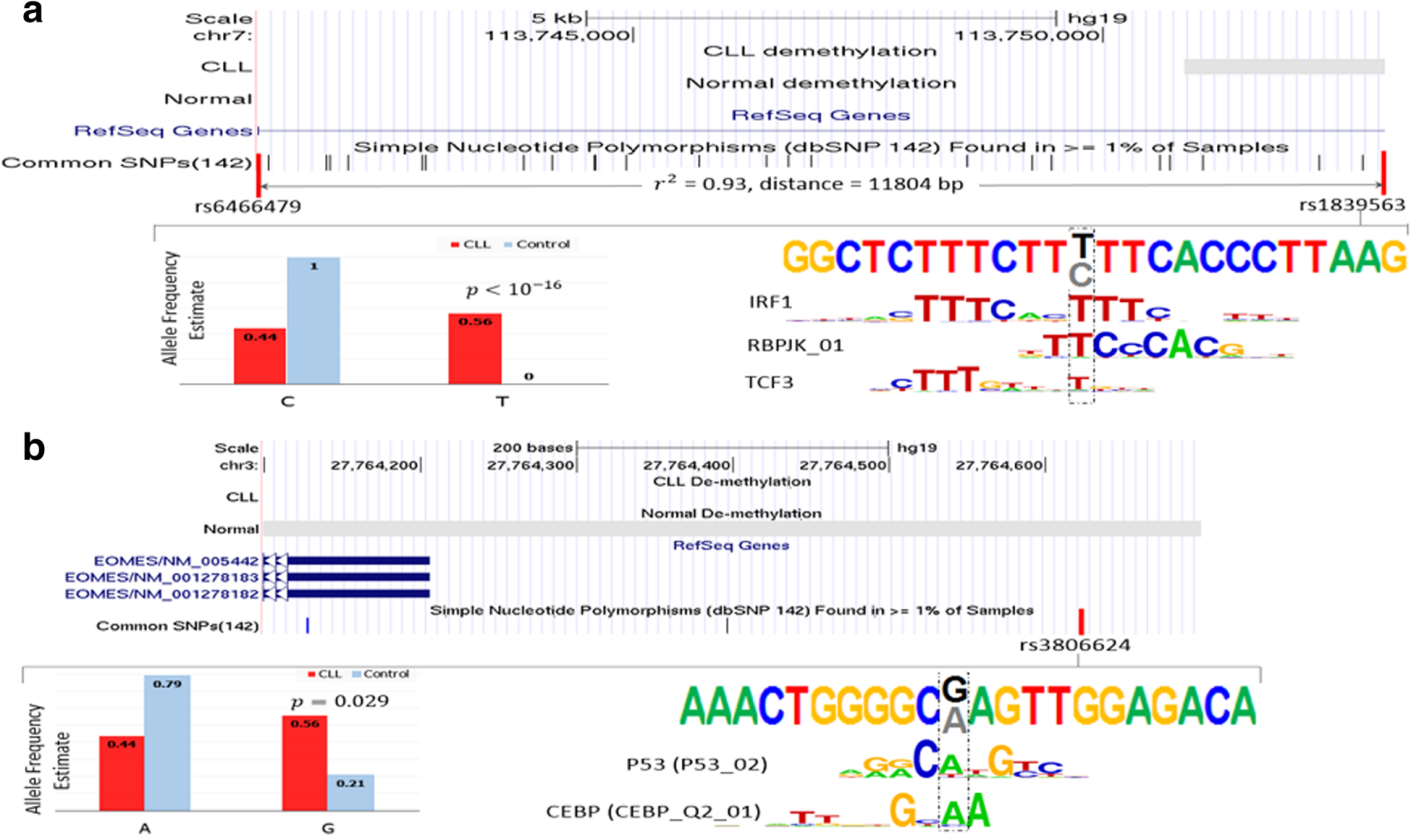
Examples of GWAS SNPs located in dREs. The dREs SNPs (**a**) rs1839563 and (**b**) rs3806624 are tightly linked to the SNPs associated with the CLL-related traits, such as haematological cancers or colorectal cancer. Bar graphs show the comparison of allele frequencies between CLL and control samples. The flanking sequences of the example SNPs are displayed, along which the example SNPs are implied by a box with dashes. For a SNP, the black allele is the one enriched in CLL (i.e., CLL-associated), while the grey allele is the one associated with normal samples. To show the TFBS change caused by CLL-associated substitutions, the TFBSs mapped to the black alleles, but not to the grey alleles, are presented for the gained-dRE SNPs (**a**), and the TFBSs mapped to the grey alleles but not black alleles are displayed in the case of the lost-dRE SNPs (**b**). Two additional examples are shown in Additional file 1: Figure S14 and S15

We next examined all cancer-related GWAS SNPs located in dREs. As an example, a gained-dRE SNP rs1839563 is strongly linked to rs6466479 (*r* ^2^ = 0.93, *distance* = 11804 *bp*), a GWAS SNP significantly associated with autoimmune disease and hematological cancers, with G being the risk allele [42]. Also, the examination of the genotype state based on RRBS-seq data (see Methods) reveals that the T allele of rs1839563 is enriched in CLL with respect to the control (binomial test *p* < 10^− 16^, see Methods, Fig. 5a). In addition, through exploring the data from the 1000 Genomes Project [20], we noticed that the allele T at rs1839563 is in a prominent haplotype with the risk allele G of GWAS SNP rs6466479 (*OR* = 257.9, Fisher’s exact test *p* = 4 × 10^− 238^, see Methods). These observations further elaborate the association of rs1839563 and its allele G with the haematological cancer. Furthermore, the mutation from C to T generates binding motifs for interferon regulatory factor 1 (IRF1), transcription factor 3 (TCF3), and RBPJk (Fig. 5a). All these TFs are over-expressed in CLL. IRF1 activates the transcription of interferons, which in turn up-regulates CD26 and CD38 in malignant B-cells [43]. RBPJk, an important regulator in the Notch signaling pathway, plays a critical role in anti-apoptotic mechanisms during CLL development [44]. TCF3, a major B-cell transcription factor also known as E2A and E47, promotes proliferation of CLL [45]. Taken together, rs1839563 demonstrates the potential association with CLL after being mutated from C to T.

Another example lies at rs3806624, a lost-dRE SNP. rs3806624 has been associated with Hodgkin’s lymphoma and has G as a risk allele in a GWAS study [46]. Our analysis shows that the allele G of rs3806624 is significantly enriched in CLL (allele frequency is 0.57 and 0.21 in CLL and control, respectively; binomial test *p* = 0.0029, Fig. 5b), indicating the possible deleteriousness of this allele in CLL. The allele substitution of G to A potentially disrupts the binding motif of P53 and CCAAT-enhancer-binding protein (CEBP) (Fig. 5b), TFs known to play roles in apoptosis and hematopoietic cell differentiation. The coincidence between the CLL-enriched allele and the reported risk allele, together with the binding disruption caused by the CLL mutation, supports the possible pathogenicity of rs3806624.

We have a total of six cancer-associated GWAS SNPs exhibiting a significant difference of allele frequency between CLL and control (Additional file 2: Table S6). Among these SNPs are the above example SNPs, rs1976684, rs2151512, rs8077394 and rs133018 (see Additional file 1: Figure S14 and S15). Most of these SNPs (5/6) exhibit a prominent haplotype association between the CLL-enriched alleles and the risk alleles detected in GWASs (Additional file 2: Table S6). These cancer SNPs, coupled with the statistical results presented in the previous section, suggest a significant association of dREs and genetic mutations inside these regions with CLL or CLL-related traits. We also observed that genetic mutations are able to change the binding sites of CLL/normal B-cell TFs, which may be the driver of phenotypic alterations.

### Changes of TFBS in CLL development

**Fig. 6:**
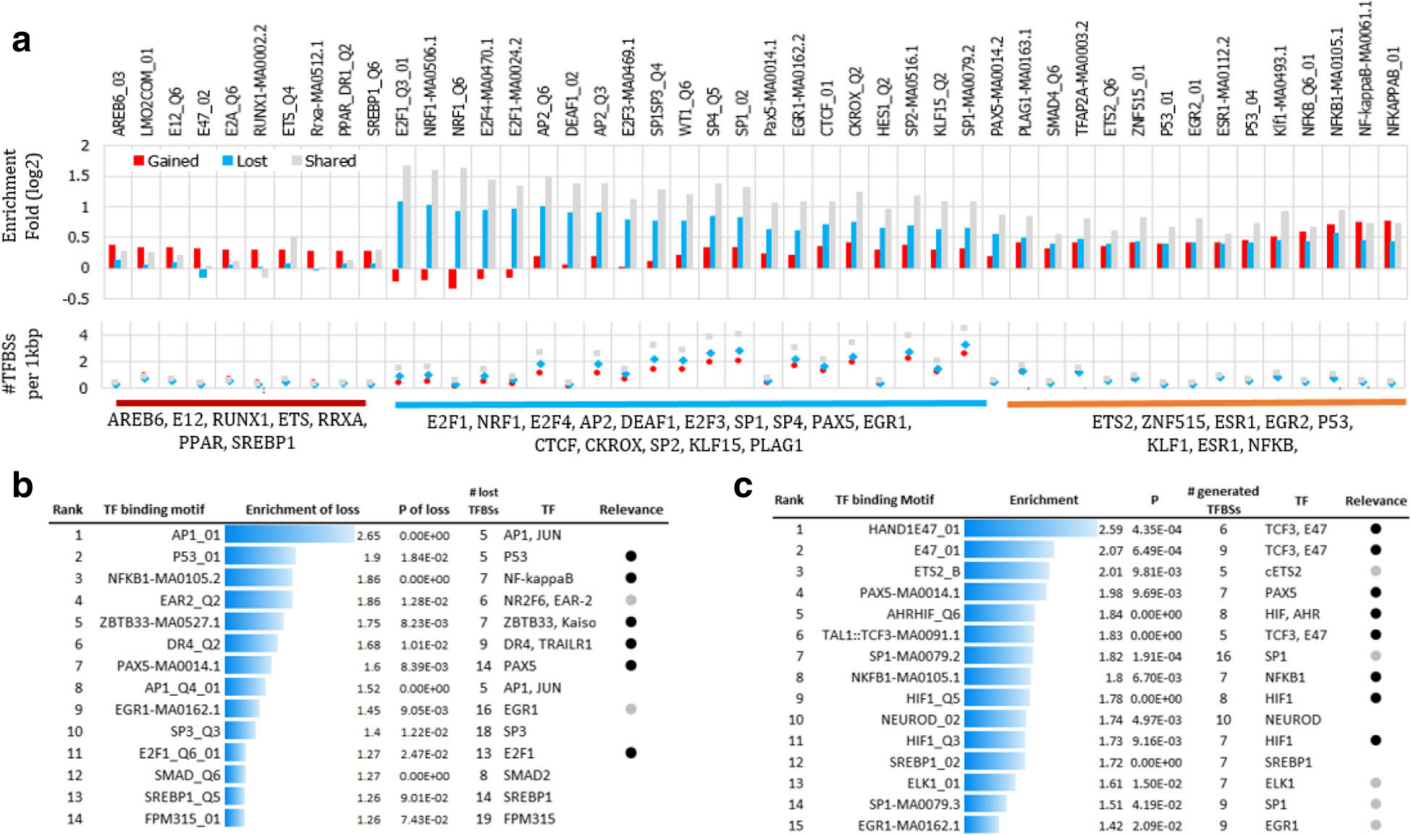
TFBS enrichment in different dRE groups. **a** Each dRE group is shown to have a distinct TFBS enrichment. **b** CLL-associated mutations in the lost dREs disrupt the binding sites of B-cell TFs. **c** CLL-associated mutations in the gained dREs generate the binding sites of CLL TFs. The black dots indicate the TFs relevant to CLL, and the grey dots represent the TFs relevant to CLL-related diseases, such as leukemia. The details are given in Additional file 2: Table S8 and S9

To investigate gene regulatory changes underlying carcinogenesis, we evaluated and contrasted transcription factor binding site (TFBS) enrichment in gained and lost dREs (see Methods). The results reveal that all gained and lost dREs display a significant enrichment of eight TFBSs (Fig. 6a), including those of NFKB1, estrogen receptor 2 (ESR1), and P53, all well-known for activating and maintaining B and CLL cells. Gained dREs are exclusively enriched for the TFBSs of TCF3 and PPAR (Fig. 6a and Additional file 2: Table S7). These TFs are major TFs in CLL and, more broadly, leukemia, as discussed above. Lost dREs are enriched with the TFBSs of PAX5, AP2, and E2F1. E2F1 has been found to be involved in tumor suppression and cell cycle, and the loss of E2F1 results in the progress of carcinogenesis and the decrease of lymphocyte tolerance [47]. PAX5 is an essential marker in the development and activation of B-cells and leukemogenesis [48]. Overall, the different TFBS signatures suggest that CLL B-cells use the distinct gene regulation pathways found in normal B-cells.

Next, we examined how genetic mutations, i.e., the allele substitutions at SNPs identified in this study, impact the binding affinity of TFs. We did not have genetic variation data directly available for the tested CLL samples. Therefore, we explored RRBS-seq data to identify SNPs strongly associated with CLL, in which the genotype in CLL samples is significantly different from the controls, along with the CLL-associated substitutions at these SNPs (see Methods). In total, 491 such SNPs were identified in dREs, of which 305 were located in the gained dREs and 186 were in lost dREs. We assessed TF binding alterations potentially caused by the CLL-associated substitutions (see Methods). By a comparison to the random positions having matched base-pair composition along the lost dREs, we noticed that the detected CLL-associated substitutions are associated with the loss of binding site of P53, NFKB2, E2F1, and PAX5 more frequently than expected (enrichment of TFBS loss > 1.5 and *p* < 0.05, Fig. 6b and Additional file 2: Table S8). E2F1 and PAX5 are the major regulators in normal B-cells, of which TFBSs have been found to be enriched uniquely along the lost dREs (Fig. 6a and Additional file 2: Table S7). Also, in the context of the gained dREs, 15 TFBSs are enriched only in the sequences carrying CLL-associated alleles (*p* < 0.01, see Methods, Fig. 6c and Additional file 2: Table S9). Most of these TFBS correspond to well-known CLL TFs, such as TCF3 and HIF1. HIF1 is required for the survival of leukemia stem cells under hypoxic environments, such as bone marrow niches [10, 49]. In addition, the CLL-association substitutions are more likely than expected to alter the binding affinity of NFKB and PAX5 in both the gained and lost dREs, compatible with the functions of these TFs in CLL as well as normal B-cells.

## Discussion and conclusion

In this study, we established a workflow to identify differentially-activated REs (dREs) in carcinogenesis and applied it to CLL data. Most of the CLL dREs are located in non-promoter gene regulatory loci, indicating a substantial role enhancer alterations play in CLL carcinogenesis. We found that dRE changes are strongly correlated with the change of gene expression, i.e., gained/lost dREs are enriched in the loci of up-/down-regulated genes in CLL, respectively.

We found that lost and gained dREs rarely co-occur in the same gene loci, suggesting reprogramming of the regulatory architecture is locus-long and not necessarily targeting individual regulatory elements in carcinogenesis. As expected, gained dREs are significantly associated with CLL-induced biological processes. For example, 68% of the genes having the function of DNA damage response exclusively harbor gained dREs, which is 2.3 times higher than expected. Also 74% of genes regulating B cell activation host gained dREs. DNA methyltransferase genes, for example, DNMT3A and MGMT, which are essential for maintaining cell cycle and methylation levels of normal B-cells, harbor multiple lost dREs but zero gained dREs in their neighborhood. In addition, both gained and lost dREs significantly coincide with CLL, lymphoma, and, more broadly, cancer-associated GWAS SNPs. Furthermore, most of the cancer-associated alleles at these SNPs (83%) are in predominant haplotypes with the risk alleles reported in GWAS. All of these findings indicate the phenotypic consequence of RE changes during CLL development.

By examining TFBS enrichment in dRE sequences, we observed that normal and CLL B-cells recruit distinct gene regulatory pathways, although both of them employ common TFs, such as NFKB and P53. Apart from these common TFs, the key TFs in normal B-cells include PAX5, E2F1 and AP2, while CLL employs TCF3, PPAR, etc. Moreover, through analyzing the impact of the identified CLL-associated mutations on TF binding, we found that these mutations change the binding activity of key TFs, i.e., disrupting/generating TF binding sites in the lost/gained dREs, more frequently than expected.

### Conclusion

Overall, through exploring sequencing data of chromatin states, we established the maps of REs in normal and cancer cells and identified genetic mutations during CLL development. The comparison between these RE maps enabled us to identify gene regulatory variations during cancer initiation in different layers, such as TF binding and chromatin interaction. To test the generalization of our pipeline, we applied it to a liver tumor dataset consisting of 4 tumor and 4 control samples [50], and noticed that the distribution of dREs is highly correlated with the change of expression of local genes (Additional file 1: Figure S16), which is similar to the finding on the CLL data analysis. This indicates that our observations are likely not limited to CLL and could be generalized to other cancers.

## Additional files

Additional file 1: Supplementary figures.
**Figure S1.** Flowchart for our data analysis. **Figure S2.** Distribution of dREs. The distributions of the distances between two nearest dREs (red) are shown for (a) gained dREs, b) lost dREs, and c) cross-class dREs (i.e., gained dREs and their nearest lost dREs). **Figure S3.** Contingency table to estimate the haplotype association between the allele 1 at a LD SNP *m* and the allele 1 at its tag GWAS SNP *m*_*tag*. **Figure S4.** Evaluation of impact of CLL substitutions on TFBS. MU, the mutant allele, is the allele enriched in CLL with respect to normal B-cells, while WT, the wild - type allele, is the allele depleted in CLL with respect to normal B-cells. **Figure S5.** Genomic distribution of the assayed CpG sites. The CpG sites located within CpG islands (CGIs) and those not in CGIs are analyzed separately. **Figure S6.** PCA of methylation levels of CpG sites located at gene regulatory regions. (a) non-promoter CpG sites and (b) promoter CpG sites. The CLL and normal samples are represented by red and grey dots, respectively. **Figure S7.** Examples of dREs and sREs in the loci of (a) IRF4 and EXOC2, (b) FOXF2 and (c) E4F1 and MLST8. sREs are marked in red bars, while gained and lost sREs are plotted in blue and green, respectively. Also promoter dREs/sREs are indicated by a black asterisk and the name of the corresponding genes. **Figure S8.** Fraction of REs (REs, lost dREs, gained dREs) and hiMRs (controls) residing in CGIs. **Figure S9.** Coverage of repeats along REs and hiMRs. **Figure S10.** Enrichment of different types of repeats in REs with respect to hiMRs. **Figure S11.** Overlap among the gene groups. Gene groups are defined according to the distribution of REs. “Shared” represents the set of genes containing the sRE(s) in their loci. Similarly “Lost” and “Gained” are the genes harboring the lost and gained dRE(s), respectively. **Figure S12.** GWAS CLL SNPs located within the detected dREs and sREs. For each SNP, GWAS association is -log10(*p* value estimated in GWAS studies). In the figures, sREs are represented by red bar, while gained and lost dREs are marked by blue and green bars, respectively. **Figure S13.** GWAS lymphoma SNPs located with the detected dREs and sREs. For each SNP, GWAS association is -log10(*p* value estimated in GWAS studies). In the figures, sREs are represented by red bar, while gained and lost dREs are marked by blue and green bars, respectively. **Figure S14.** rs1976684, a SNP residing in a lost dRE, is in an LD block (*p* ^2^ = 1.0, *distance* = 2564 *bp*) with rs501764, a GWAS SNP significantly associated with Hodgkin’s lymphoma [1] (Figure S13). The allele G of rs501764 is in a prominent haplotype (*OR* = 432.6, Fisher’s exact test *p* = 2 × 10^− 133^) with the allele G at rs1976684, the pathogenic allele for Hodgkin’s lymphoma [1]. Furthermore, the allele G at rs1976684 recurs significantly in CLL samples as compared to controls (*p* = 2 × 10^− 10^). Another line of evidence is that rs1976684 has a strong linkage (*r* ^2^ = 1.0) with rs4143094, a colorectal-cancer SNP with the risk allele of T [2]. Also, the disease allele T at rs4143094 is in a significant haplotype with the CLL-rich allele G at rs1976684 (*OR* = 70.7, Fisher’s exact test *p* = 3 × 10^− 252^). Collectively, a lost-dRE SNP rs1976684 is significantly linked to two GWAS SNPs associated with cancers, including lymphoma, a haematological cancer. The CLL-enriched allele of rs1976684 significantly co-occurs with the risk alleles of these GWAS SNPs. Moreover, the mutation from A to G at rs19766684 results in the loss of binding motifs of nuclear receptor subfamily 2 group F member 1 (NR2F1), a TF found to play a crucial role in development and differentiation processes in B-cell [3], further suggesting that rs1976684 is a potential CLL SNP with G as the culprit allele. **Figure S15.** rs211512, a cancer-associated gained-dRE SNP. rs211512 has a strong LD to rs4925386 (*r* ^2^ = 1.0, *distance* = 7549 *bp*), a colorectal-cancer GWAS SNP [4]. Its over-represented allele C (*p* < 10^− 16^) is in a significant haplotype with the cancer-risk allele at rs12193698 (*OR* = 1482.16, Fisher’s exact test *p* < 10^− 300^). All of these suggest the cancer-association of rs2151512 and its allele C, which is further supported by the observation that the CLL mutation at rs2151512 (replacing T with C) generates the binding motifs for GFI1B. GFI1B is a well-recognized major regulator of early hematopoiesis and hematopoietic stem cells, and has been associated with human blood diseases, including leukemia and lymphoma [5, 6]. The black allele is the one enriched in CLL (i.e., CLL-associated), while the grey allele is the one associated with normal samples. To show the TFBS change caused by this gained dRE SNP, the TFBS exclusively mapped to the black allele is presented here. **Figure S16.** The results of liver tumor dataset. This dataset consists of DNA methylation profiles of 4 tumor and 4 control samples (Gene Expression Omnibus, GSE70090, [7]). We detected 51988 gained dREs, 22948 lost dREs and 12476 sREs. The gained and lost dREs are enriched around the genes up and down-regulated in liver tumor, respectively. ** means binomial test *p* < 0.0001, while * is for the case of *p <*0.05. (DOCX 987 kb)
Additional file 2: Supplementary tables.
**Table S1.** Overlap and enrichment of repeated elements along dREs. **Table S2.** Enrichment of dREs in the regulatory domains of CLL-up/down-regulated genes. **Table S3.** Enrichment of gene groups to Gene Ontology biological processes. **Table S4.** Density of GWAS SNPs associated with agglomerated traits along dRE. **Table S5.** Association of dREs to individual GWAS traits. **Table S6.** Prediction results and GWAS knowledge of the cancer-related GWAS SNPs which exhibit the significant allele frequency difference between CLL and normal samples. **Table S7.** Enrichment of TFBSs along dREs. **Table S8.** Enrichment of TFBS loss caused by CLL-associated alleles along lost dREs. **Table S9.** Enrichment of TFBS gain caused by CLL-associated alleles along gained dREs. (XLSX 64 kb)

## Abbreviations

CLL: Chronic lymphocytic leukemia
dRE: RE differently activated during cancer development
hiMR: Highly-methylated regions across different cells
RE: Regulatory element
sRE: Regulatory element shared by normal and cancer cells
