# Supplementary material for: Epigenetic and genetic alterations and their influence on gene regulation in chronic lymphocytic leukemia
Source: BMC Genomics. 2017 Mar 16;18:236. doi: 10.1186/s12864-017-3617-6 (PMC5353786; doi:10.1186/s12864-017-3617-6)
Supplement: Additional file 1: Supplementary figures. — Figure S1. Flowchart for our data analysis. Figure S2. Distribution of dREs. The distributions of the distances between two nearest dREs (red) are shown for (a) gained dREs, b) lost dREs, and c) cross-class dREs (i.e., gained dREs and their nearest lost dREs). Figure S3. Contingency table to estimate the haplotype association between the allele 1 at a LD SNP m and the allele 1 at its tag GWAS SNP m_tag. Figure S4. Evaluation of impact of CLL substitutions on TFBS. MU, the mutant allele, is the allele enriched in CLL with respect to normal B-cells, while WT, the wild - type allele, is the allele depleted in CLL with respect to normal B-cells. Figure S5. Genomic distribution of the assayed CpG sites. The CpG sites located within CpG islands (CGIs) and those not in CGIs are analyzed separately. Figure S6. PCA of methylation levels of CpG sites located at gene regulatory regions. (a) non-promoter CpG sites and (b) promoter CpG sites. The CLL and normal samples are represented by red and grey dots, respectively. Figure S7. Examples of dREs and sREs in the loci of (a) IRF4 and EXOC2, (b) FOXF2 and (c) E4F1 and MLST8. sREs are marked in red bars, while gained and lost sREs are plotted in blue and green, respectively. Also promoter dREs/sREs are indicated by a black asterisk and the name of the corresponding genes. Figure S8. Fraction of REs (REs, lost dREs, gained dREs) and hiMRs (controls) residing in CGIs. Figure S9. Coverage of repeats along REs and hiMRs. Figure S10. Enrichment of different types of repeats in REs with respect to hiMRs. Figure S11. Overlap among the gene groups. Gene groups are defined according to the distribution of REs. “Shared” represents the set of genes containing the sRE(s) in their loci. Similarly “Lost” and “Gained” are the genes harboring the lost and gained dRE(s), respectively. Figure S12. GWAS CLL SNPs located within the detected dREs and sREs. For each SNP, GWAS association is -log10(p value estimated in GWAS studies). In the figures, sRE [file 12864_2017_3617_MOESM1_ESM.docx]

**Supplementary materials**

**Epigenetic and genetic alterations and their influence on gene regulation in CLL**

**
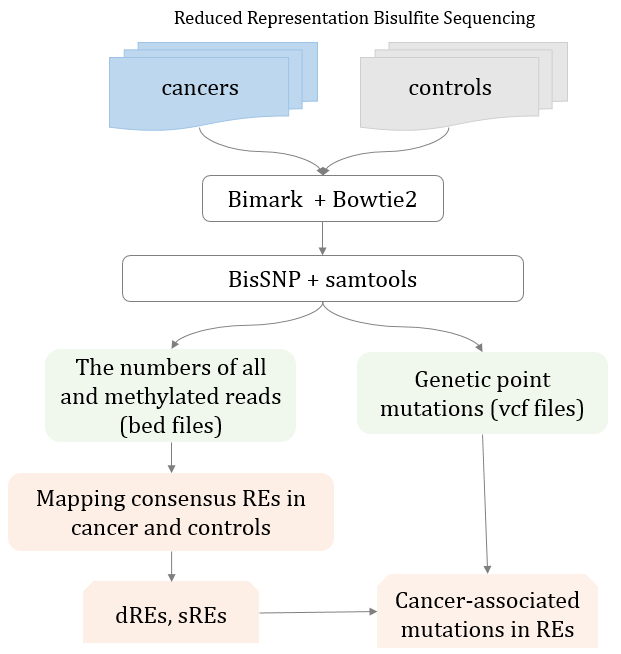
**

**Figure S1** Flowchart for our data analysis.


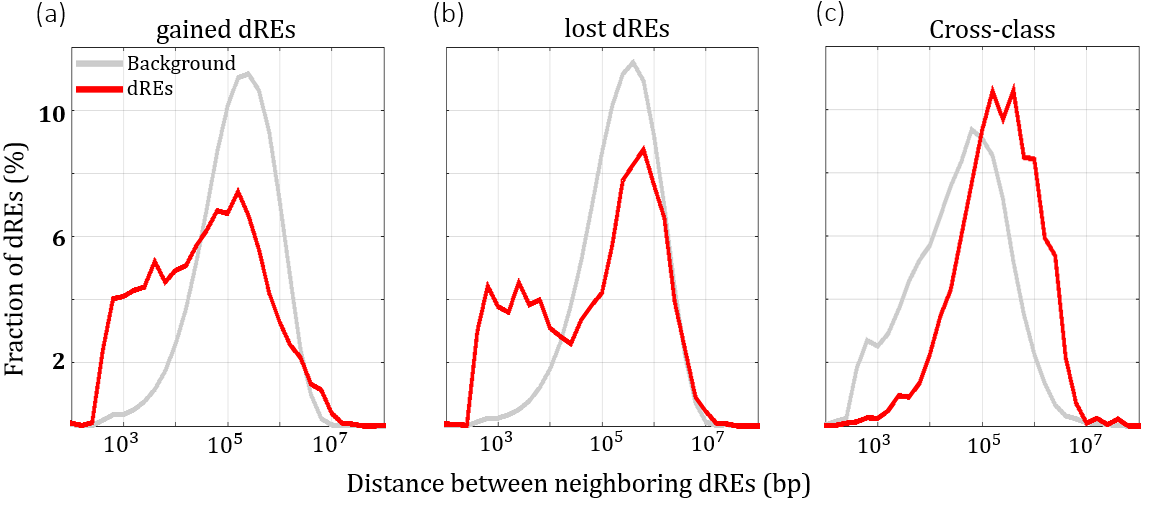


**Figure S2** Distribution of dREs. The distributions of the distances between two nearest dREs (red) are shown for (a) gained dREs, b) lost dREs, and c) cross-class dREs (i.e., gained dREs and their nearest lost dREs).


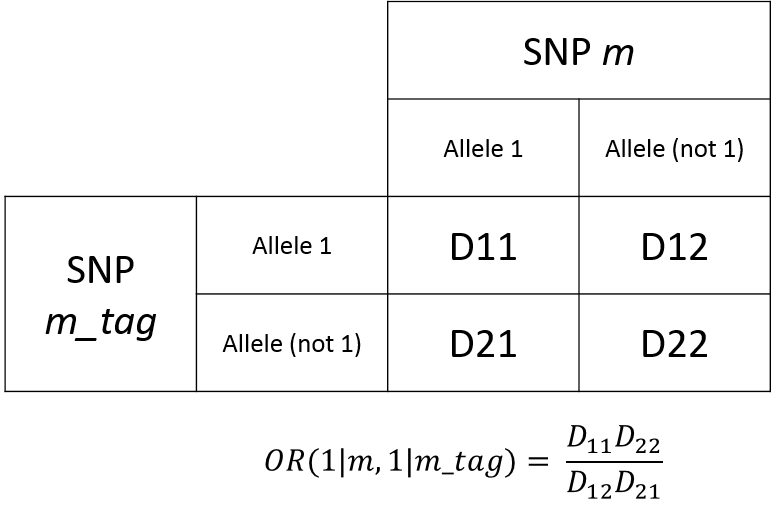


**Figure S3** Contingency table to estimate the haplotype association between the allele 1 at a LD SNP $m$ and the allele 1 at its tag GWAS SNP $m\_tag$.


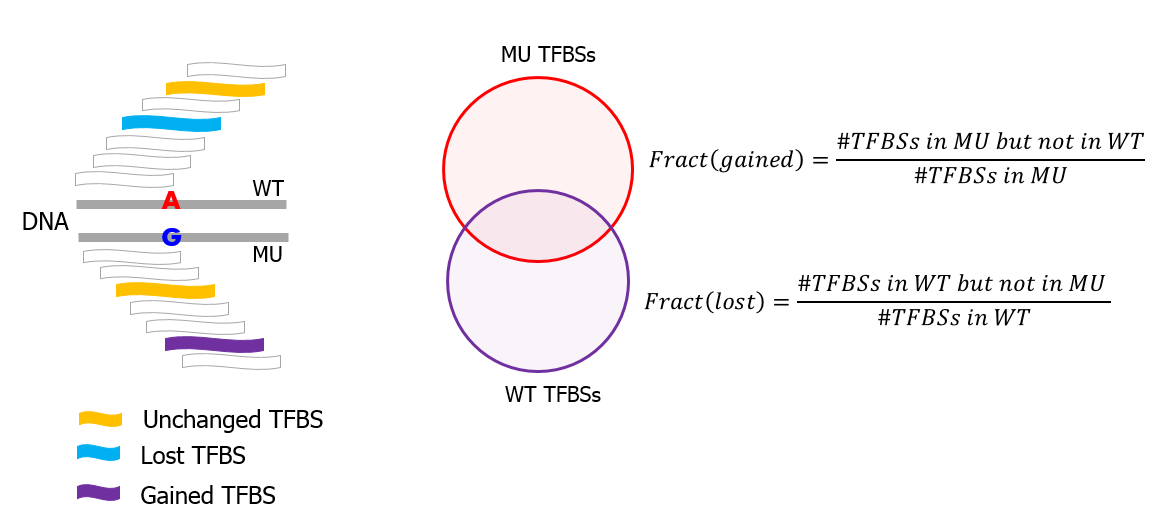


**Figure S4** Evaluation of impact of CLL substitutions on TFBS. MU, the mutant allele, is the allele enriched in CLL with respect to normal B-cells, while WT, the wild type allele, is the allele depleted in CLL with respect to normal B-cells.


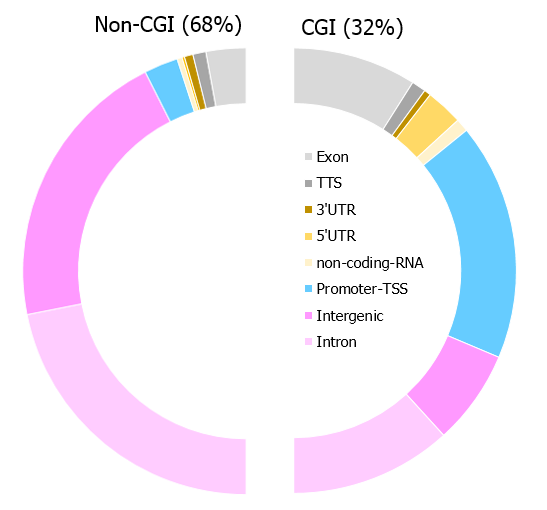


**Figure S5** Genomic distribution of the assayed CpG sites. The CpG sites located within CpG islands (CGIs) and those not in CGIs are analyzed separately.


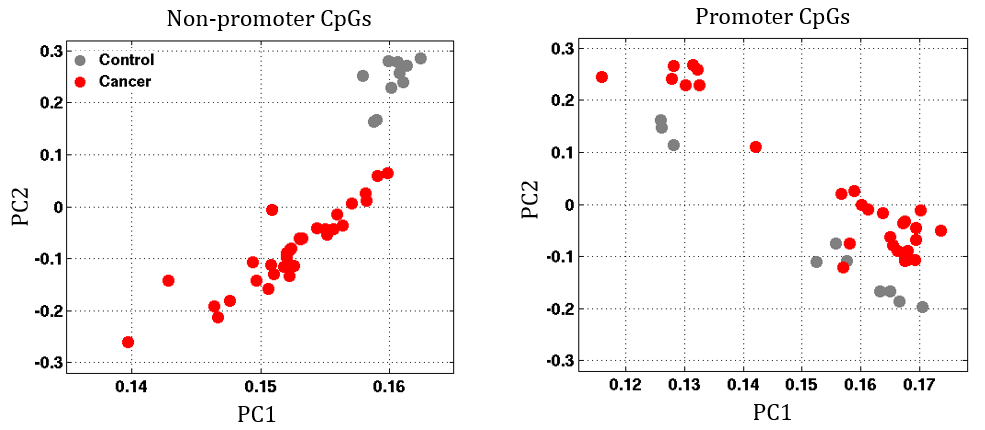


**Figure S6** PCA of methylation levels of CpG sites located at gene regulatory regions. (a) non-promoter CpG sites and (b) promoter CpG sites. The CLL and normal samples are represented by red and grey dots, respectively.


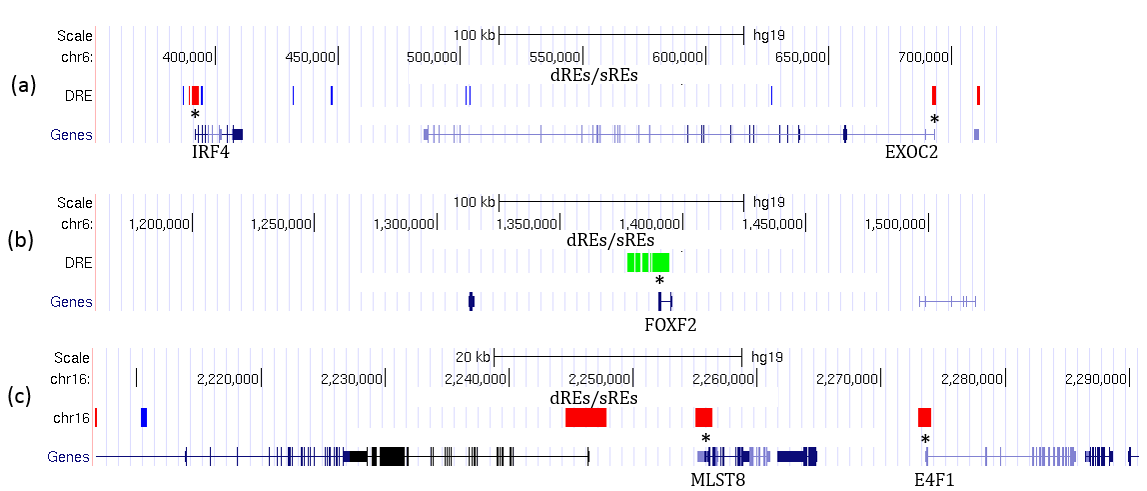


**Figure S7** Examples of dREs and sREs in the loci of (a) IRF4 and EXOC2, (b) FOXF2 and (c) E4F1 and MLST8. sREs are marked in red bars, while gained and lost sREs are plotted in blue and green, respectively. Also promoter dREs/sREs are indicated by a black asterisk and the name of the corresponding genes.


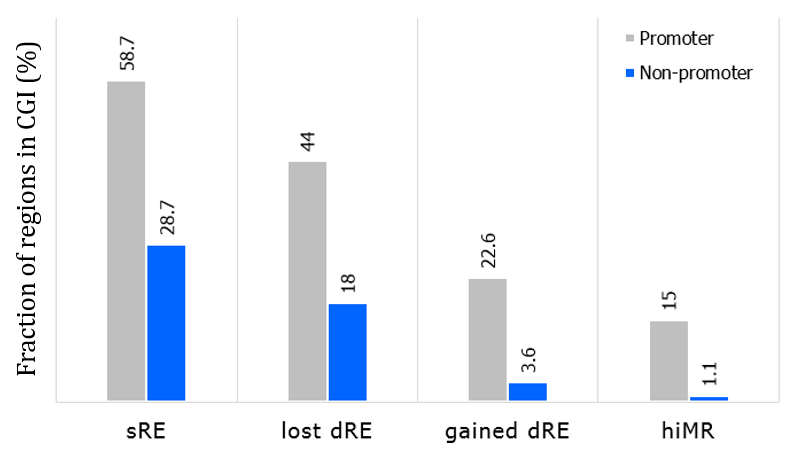


**Figure S8** Fraction of REs (REs, lost dREs, gained dREs) and hiMRs (controls) residing in CGIs.


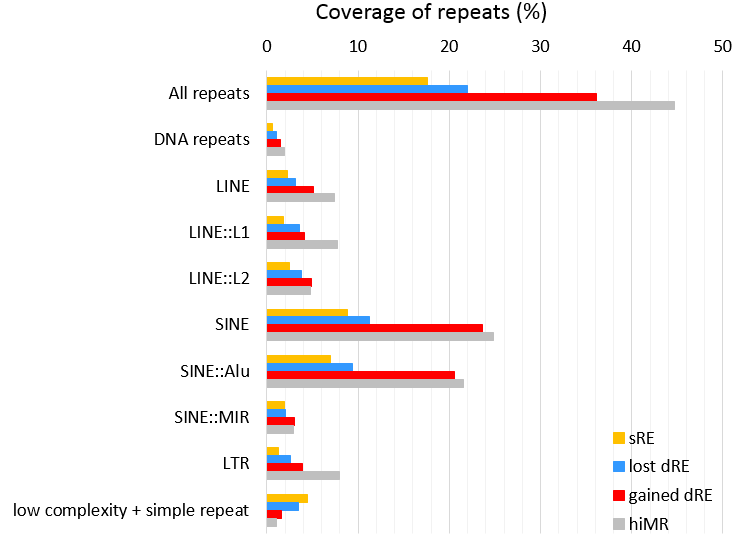


**Figure S9** Coverage of repeats along REs and hiMRs.


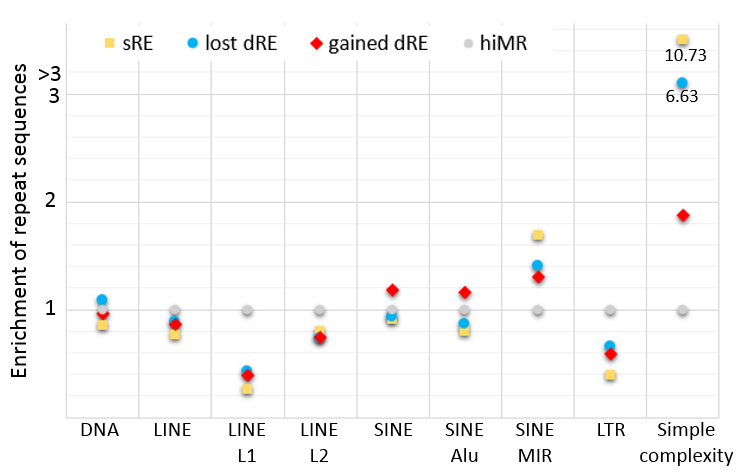


**Figure S10** Enrichment of different types of repeats in REs with respect to hiMRs.


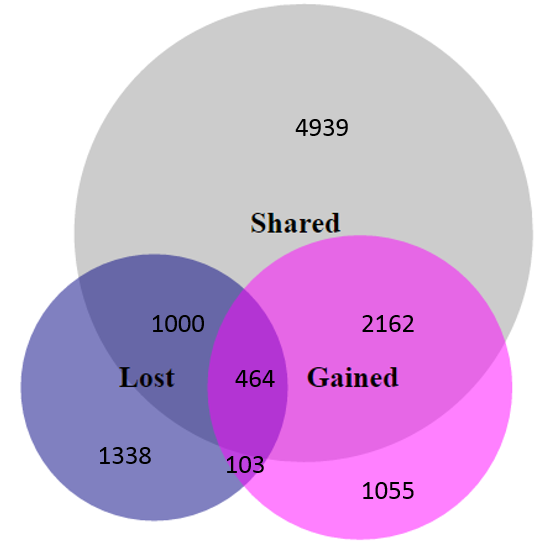


**Figure S11** Overlap among the gene groups. Gene groups are defined according to the distribution of REs. “Shared” represents the set of genes containing the sRE(s) in their loci. Similarly “Lost” and “Gained” are the genes harboring the lost and gained dRE(s), respectively.


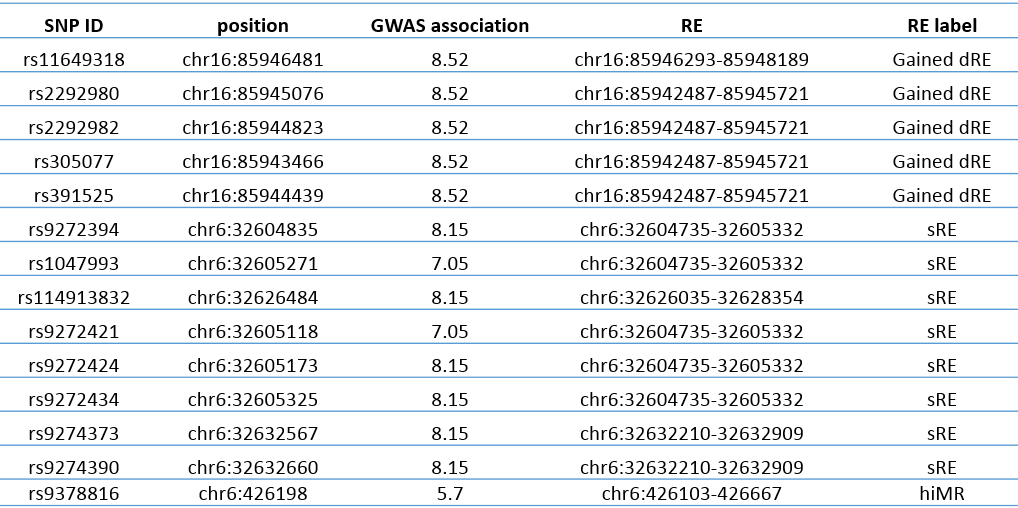


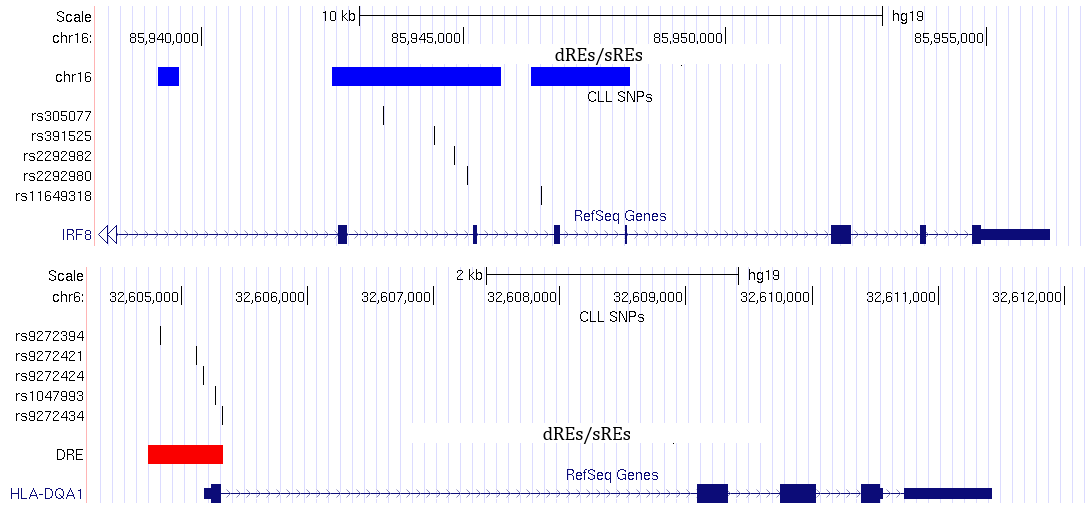


**Figure S12** GWAS CLL SNPs located within the detected dREs and sREs. For each SNP, GWAS association is -log10(pvalue estimated in GWAS studies). In the figures, sREs are represented by red bar, while gained and lost dREs are marked by blue and green bars, respectively.


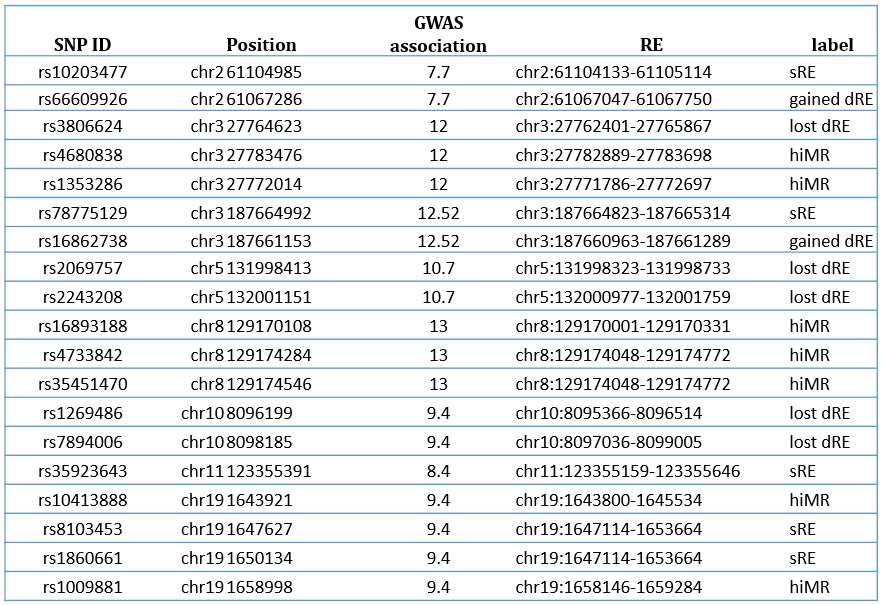


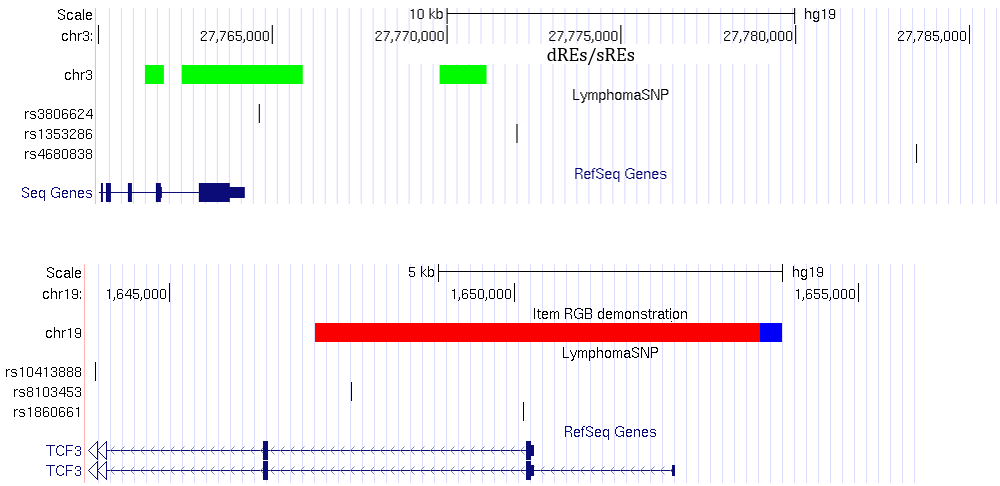


**Figure S13** GWAS lymphoma SNPs located with the detected dREs and sREs. For each SNP, GWAS association is -log10(pvalue estimated in GWAS studies). In the figures, sREs are represented by red bar, while gained and lost dREs are marked by blue and green bars, respectively.


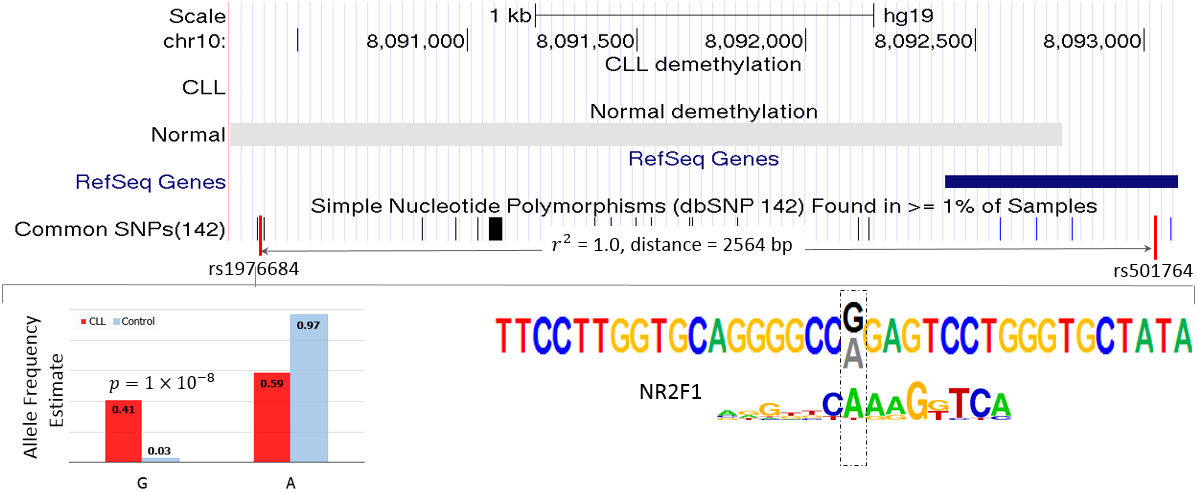


**Figure S14** rs1976684, a SNP residing in a lost dRE, is in an LD block ($p^{2}=1.0, distance=2564 bp$) with rs501764, a GWAS SNP significantly associated with Hodgkin’s lymphoma [[1](#_ENREF_1)] (Fig. S13). The allele G of rs501764 is in a prominent haplotype ($OR=432.6$, Fisher’s exact test$p=2\times{10}^{-133}$) with the allele G at rs1976684, the pathogenic allele for Hodgkin’s lymphoma [[1](#_ENREF_1)]. Furthermore, the allele G at rs1976684 recurs significantly in CLL samples as compared to controls ($p=2\times{10}^{-10}$ ). Another line of evidence is that rs1976684 has a strong linkage ($r^{2}=1.0$) with rs4143094, a colorectal-cancer SNP with the risk allele of T [[2](#_ENREF_2)]. Also, the disease allele T at rs4143094 is in a significant haplotype with the CLL-rich allele G at rs1976684 ($OR=70.7$, Fisher’s exact test$p=3\times{10}^{-252}$). Collectively, a lost-dRE SNP rs1976684 is significantly linked to two GWAS SNPs associated with cancers, including lymphoma, a haematological cancer. The CLL-enriched allele of rs1976684 significantly co-occurs with the risk alleles of these GWAS SNPs. Moreover, the mutation from A to G at rs19766684 results in the loss of binding motifs of nuclear receptor subfamily 2 group F member 1 (NR2F1) (Fig. 13), a TF found to play a crucial role in development and differentiation processes in B-cell [[3](#_ENREF_3)], further suggesting that rs1976684 is a potential CLL SNP with G as the culprit allele.


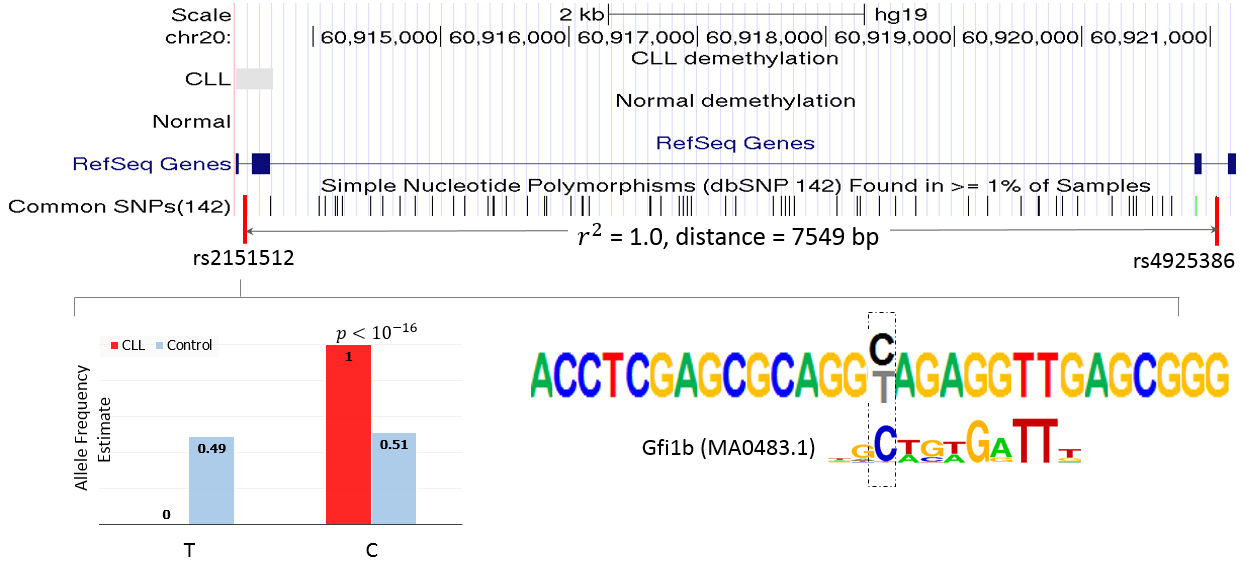


**Figure S15** rs211512, a cancer-associated gained-dRE SNP. rs211512 has a strong LD to rs4925386 ($r^{2}=1.0, distance=7549 bp$), a colorectal-cancer GWAS SNP [[4](#_ENREF_4)]. Its over-represented allele C ($p<{10}^{-16}$) is in a significant haplotype with the cancer-risk allele at rs12193698 ($OR=1482.16$, Fisher’s exact test$p<{10}^{-300}$). All of these suggest the cancer-association of rs2151512 and its allele C, which is further supported by the observation that the CLL mutation at rs2151512 (replacing T with C) generates the binding motifs for GFI1B. GFI1B is a well-recognized major regulator of early hematopoiesis and hematopoietic stem cells, and has been associated with human blood diseases, including leukemia and lymphoma [[5](#_ENREF_5), [6](#_ENREF_6)]. The black allele is the one enriched in CLL (i.e., CLL-associated), while the grey allele is the one associated with normal samples. To show the TFBS change caused by this gained dRE SNP, the TFBS exclusively mapped to the black allele is presented here.

**
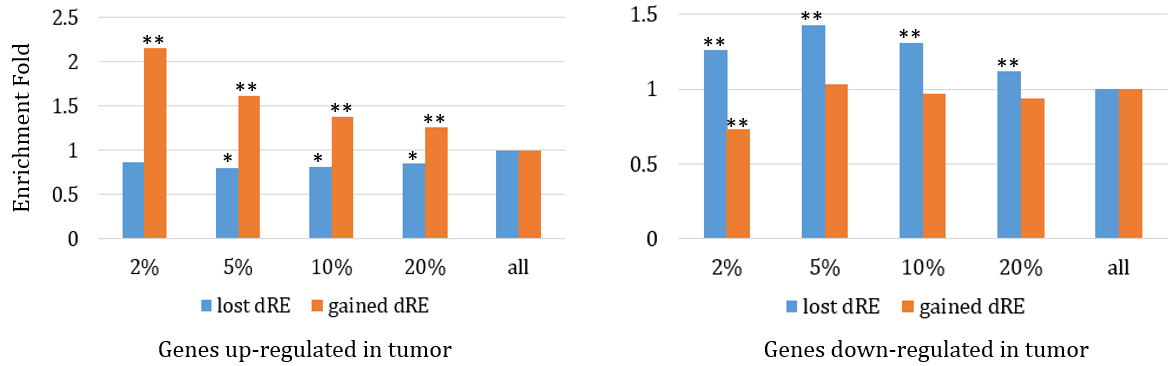
**

**Figure S16** The results of liver tumor dataset. This dataset consists of DNA methylation profiles of 4 tumor and 4 control samples (Gene Expression Omnibus, GSE70090, [[7](#_ENREF_7)]). We detected 51988 gained dREs, 22948 lost dREs and 12476 sREs. The gained and lost dREs are enriched around the genes up and down-regulated in liver tumor, respectively. ** means binomial test $p<0.0001$, while * is for the case of $p<0.05$.

**Reference**

1. Frampton, M., et al., *Variation at 3p24.1 and 6q23.3 influences the risk of Hodgkin’s lymphoma.* Nat Commun, 2013. **4**.

2. Figueiredo, J.C., et al., *Genome-Wide Diet-Gene Interaction Analyses for Risk of Colorectal Cancer.* PLoS Genetics, 2014. **10**(4): p. e1004228.

3. Xu, M., et al., *The role of the orphan nuclear receptor COUP-TFII in tumorigenesis.* Acta Pharmacol Sin, 2015. **36**(1): p. 32-36.

4. Houlston, R.S., et al., *Meta-analysis of three genome-wide association studies identifies susceptibility loci for colorectal cancer at 1q41, 3q26.2, 12q13.13 and 20q13.33.* Nat Genet, 2010. **42**(11): p. 973-977.

5. Möröy, T., et al., *From cytopenia to leukemia: the role of Gfi1 and Gfi1b in blood formation.* Blood, 2015. **126**(24): p. 2561-2569.

6. Koldehoff, M., et al., *Additive antileukemia effects by GFI1B- and BCR-ABL-specific siRNA in advanced phase chronic myeloid leukemic cells.* Cancer Gene Ther, 2013. **20**(7): p. 421-427.

7. Li, X., et al., *Whole-genome analysis of the methylome and hydroxymethylome in normal and malignant lung and liver.* Genome Research, 2016. **26**(12): p. 1730-1741.
